# CD8^+^ T cells sustain antitumor response by mediating crosstalk between adenosine A2A receptor and glutathione/GPX4

**DOI:** 10.1172/JCI170071

**Published:** 2024-03-05

**Authors:** Siqi Chen, Jie Fan, Ping Xie, Jihae Ahn, Michelle Fernandez, Leah K. Billingham, Jason Miska, Jennifer D. Wu, Derek A. Wainwright, Deyu Fang, Jeffrey A. Sosman, Yong Wan, Yi Zhang, Navdeep S. Chandel, Bin Zhang

**Affiliations:** 1 Department of Medicine, Hematology/Oncology Division, Robert H. Lurie Comprehensive Cancer Center,; 2 Department of Neurological Surgery,; 3 Department of Urology, and; 4 Department of Pathology, Northwestern University Feinberg School of Medicine, Chicago, Illinois, USA.; 5 Department of Pharmacology and Chemical Biology, Emory University School of Medicine, Atlanta, Georgia, USA.; 6 Biotherapy Center, The First Affiliated Hospital of Zhengzhou University, Zhengzhou, Henan, China.; 7 Department of Medicine; Pulmonary and Critical Care Division, and; 8 Department of Biochemistry and Molecular Genetics, Northwestern University Feinberg School of Medicine, Chicago, Illinois, USA.

**Keywords:** Immunology, Metabolism, Cancer immunotherapy, T cells

## Abstract

Antitumor responses of CD8^+^ T cells are tightly regulated by distinct metabolic fitness. High levels of glutathione (GSH) are observed in the majority of tumors, contributing to cancer progression and treatment resistance in part by preventing glutathione peroxidase 4–dependent (GPX4-dependent) ferroptosis. Here, we show the necessity of adenosine A2A receptor (A2AR) signaling and the GSH/GPX4 axis in orchestrating metabolic fitness and survival of functionally competent CD8^+^ T cells. Activated CD8^+^ T cells treated ex vivo with simultaneous inhibition of A2AR and lipid peroxidation acquire a superior capacity to proliferate and persist in vivo, demonstrating a translatable means to prevent ferroptosis in adoptive cell therapy. Additionally, we identify a particular cluster of intratumoral CD8^+^ T cells expressing a putative gene signature of GSH metabolism (GMGS) in association with clinical response and survival across several human cancers. Our study addresses a key role of GSH/GPX4 and adenosinergic pathways in fine-tuning the metabolic fitness of antitumor CD8^+^ T cells.

## Abstract 2

Activated CD8+ T cells treated with A2AR inhibition and lipid peroxidation proliferate in vivo, demonstrating a translatable means to prevent ferroptosis in adoptive cell therapy.

## Graphical abstract

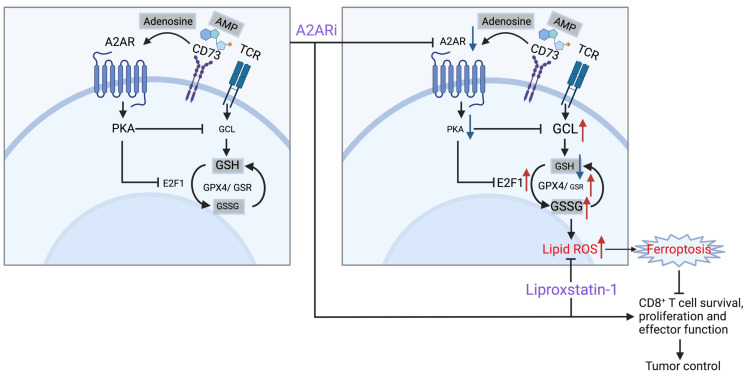

## Introduction

Glutathione (GSH) is the most abundant antioxidant that maintains cellular redox homeostasis. De novo synthesis of GSH is coordinated by the sequential actions of glutaminase, γ-glutamate-cysteine ligase, and GSH synthetase, as well as regeneration from glutathione disulfide (GSSG) by glutathione disulfide reductase (GSR) (1). GSH is oxidized to GSSG through glutathione peroxidases (GPXs) to buffer reactive oxygen species (ROS). The ratio of GSH to GSSG decreases with exposure to oxidative stress (2, 3), and an imbalance in the GSH/GSSG ratio has been documented in a variety of disorders, such as cancer, neurodegenerative diseases, and viral infections (4). Indeed, high levels of GSH are observed in the majority of tumors, contributing to cancer progression and treatment resistance in part by preventing glutathione peroxidase 4–dependent (GPX4-dependent) ferroptosis, an iron-dependent programmed cell death (5). Therefore, targeting GPX4 (6, 7) and/or GSH synthesis (8) to induce cancer cell ferroptosis has become an attractive means for cancer therapy (9).

Antitumor responses of CD8^+^ T cells are tightly regulated by distinct metabolic fitness (10–15). Histologically, T lymphocyte activation (16) and proliferation (17) are directly dependent on intracellular GSH availability. Previous studies have shown that GSH biosynthesis is required for inflammatory T cell responses (18, 19) and regulatory T cell suppressive function (20). Moreover, optimal CD8^+^ T cell function requires GPX4 (21). In addition, others have reported that an increased antioxidative capacity of CD8^+^ T cells is crucial for a better antitumor response (22, 23). However, the molecular mechanism of the GSH/GPX4 metabolic axis in modulating the antitumor response of CD8^+^ T cells still remains elusive. In this study, we confirmed that GSH availability was required for the survival, expansion, and effector function of cytotoxic CD8^+^ T cells. GPX4 deficiency in T cells significantly accelerated the tumor growth accompanied with diminished intratumoral T cell accumulation. Adoptive cell therapy (ACT) using activated CD8^+^ T cells with genetic deletion of GPX4 also failed to enable antitumor immunity and subsequent tumor control. Mechanistically, inactivation of the CD73/adenosine A2A receptor (A2AR) signaling cascade accelerated the de novo synthesis and consumption of GSH with GSSG accumulation and concomitant mitochondrial lipid peroxidation, which linked potentially to PKA/E2F1–dependent fine-tuning of the ratio of GSR to GPX4. Furthermore, activated CD8^+^ T cells treated with A2AR inhibitor (A2ARi) plus liproxstatin-1 (Lip-1), a potent inhibitor of lipid peroxidation ex vivo, acquired a superior capacity to proliferate and persist in vivo, and promoted antitumor efficacy of ACT. Additionally, a unique cluster of intratumoral CD8^+^ T cells expressing a putative gene signature of GSH metabolism (GMGS) was associated with clinical response and survival across several human cancers. Given the seminal discovery of the critical and non-redundant immunosuppressive role of A2AR (24) and the therapeutic potential of A2AR blockade for T cell–based immunotherapy (25), our study demonstrates a viable strategy by targeting A2AR in combination with inhibition of lipid peroxidation to improve persistence and functionality of ACT against cancer.

## Results

### Intracellular GSH sustains the antitumor response of CD8^+^ T cells through GPX4.

**Figure 1:**
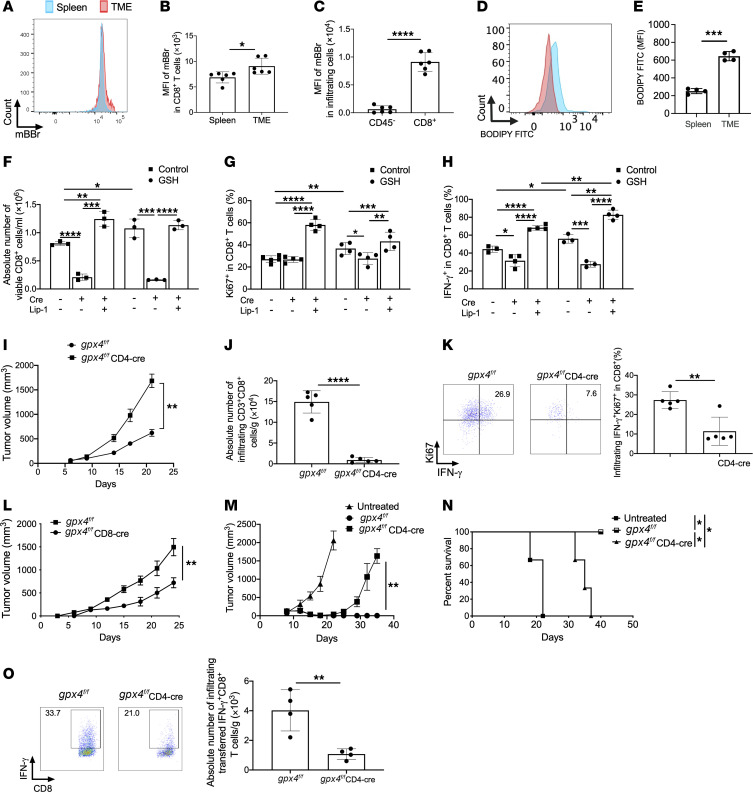
The GSH/GPX4 axis is required for antitumor immune responses of activated CD8^+^ T cells. (**A**–**C**) Intracellular GSH content indicated by mBBr staining was measured in splenic CD8^+^ cells and tumor-infiltrating CD8^+^ and CD45^–^ cells from LLC1-OVA–bearing mice. (**D** and **E**) Lipid ROS level indicated by BODIPY FITC was measured in splenic CD8^+^ cells and tumor-infiltrating CD8^+^ T cells from LLC1-OVA–bearing mice. (**F**–**H**) *gpx4^fl/fl^* and *gpx4^fl/fl^ CD4^cre^* OT-1 CD8^+^ T cells were activated in the presence of 10 ng/mL OVA-I and 1 μg/mL anti-CD28 with or without cell-permeable GSH, Lip-1, or a combination of both. On day 6, the percentages and/or numbers of viable (**F**), Ki67^+^ (**G**), and IFN-γ^+^ (**H**) T cells were measured. (**I**–**K**) *gpx4^fl/fl^* and *gpx4^fl/fl^ CD4^cre^* hosts were inoculated with MC38 tumor cells (*n* = 5). Tumor volume was measured twice a week (**I**). On day 21, the tumors were harvested, and tumor-infiltrating CD8^+^ T cells were counted (**J**) and evaluated for IFN-γ production by flow cytometry (**K**). (**L**) Tumor growth was measured in MC38-bearing *gpx4^fl/fl^* and *gpx4^fl/fl^ CD8^cre^* mice (*n* = 5). (**M**–**O**) Activated *gpx4^fl/fl^* or *gpx4^fl/fl^ CD4^cre^* OT-1 CD8^+^ T cells were transferred i.v. into EG7-bearing mice. Tumor growth (**M**) and survival (**N**) were measured (*n* = 3). Seven days after T cell transfer, tumor tissues were collected for detection of transferred OT-1 CD8^+^IFN-γ^+^ T cells (**O**). Results are representative of more than 3 (**A**–**C** and **F**–**H**) or 2 (**D**, **E**, **I**–**K**, and **M**–**O**) independent experiments. Data were analyzed by 2-tailed *t* test (**B**, **C**, **E**, **J**, **K**, and **O**), 2-way ANOVA (**F**–**H**, **I**, **L**, and **M**), and log-rank test for survival curves (**N**). Data plotted are mean ± SEM from biological replicates. *P < 0.05, **P < 0.01, ***P < 0.001, ****P < 0.0001.

We confirmed that monobromobimane (mBBr) can efficiently indicate the intracellular GSH in CD8^+^ T cells, as the MFI of mBBr is highly correlated with the GSH content detected by GSH/GSSG-Glo assay (Promega Corp.) from the same cohort of cells (Supplemental Figure 1A; supplemental material available online with this article; https://doi.org/10.1172/JCI170071DS1). Then we found a significantly increased level of intracellular GSH in activated CD8^+^ T cells after T cell antigen receptor (TCR) stimulation as indicated by mBBr staining (Supplemental Figure 1B). Within the tumor microenvironment, CD8^+^ T cells contained higher levels of GSH than splenic counterparts from tumor-bearing mice (Figure 1, A and B, and Supplemental Figure 1C). Infiltrating CD8^+^ T cells showed more abundant GSH than tumor cells (Figure 1C). On the other hand, infiltrating CD8^+^ T cells also displayed significantly much higher levels of lipid ROS than splenic counterparts (Figure 1, D and E), suggesting that the balance between GSH and lipid ROS may contribute to their function. To better understand how changes in intracellular GSH metabolism might contribute to the expansion, survival, and function of CD8^+^ T cells upon “chronic” antigenic stimulation, we used an in vitro system (26) whereby activated transgenic or wild-type (WT) T cells were expanded in the presence of persistent antigenic stimulation with either specific antigen (OVA-I/gp100) or anti-CD3–mediated stimulation and anti-CD28 costimulation for 7 days. The GSH synthesis inhibitor buthionine sulfoximine (BSO) was added. Notably, the number of viable activated CD8^+^ T cells upon BSO treatment decreased significantly (Supplemental Figure 1D), and these cells showed little proliferative capacity as indicated by Ki67 expression (Supplemental Figure 1E) and lower IFN-γ production (Supplemental Figure 1F). The addition of GSH restored the number, proliferation, and function of CD8^+^ T cells, even in the presence of BSO (Supplemental Figure 1, D–F). These results show that GSH is required for CD8^+^ T cell accumulation and function.

In mammals, GPX family members can utilize GSH as a reducing agent in combating oxidative stress and maintaining redox balance via a different mechanism of action and site of action. Unlike other GPXs, GPX4, the only enzyme that directly reduces and destroys lipid hydroperoxides, has been recently reported to maintain CD8^+^ T cell survival and function (21). However, the processes underlying the metabolic action of the GSH/GPX4 axis in antitumor CD8^+^ T cell fate and function for therapy are not understood at mechanistic depth. In this regard, we crossed *gpx4^fl/fl^* mice with CD4-cre mice to generate conditional knockout mice with T cell–specific depletion of GPX4 (CD4-cre^+^ *gpx4^fl/fl^* [GPX4 cKO]) for further investigation. GPX4 deficiency in CD8^+^ T cells resulted in a lesser consumption of intracellular GSH (Supplemental Figure 1G) accompanied by an increase in lipid ROS level (Supplemental Figure 1H) and a decrease in cellular viability (Supplemental Figure 1, I and J). Consistently, the addition of exogenous GSH failed to restore cell survival, further suggesting an essential contribution of GPX4 to the GSH-mediated effect (Figure 1, F–H). As GPX4 deficiency results in the accumulation of lipid peroxidation and subsequent ferroptosis of T cells (21), treatment with Lip-1 greatly decreased lipid ROS (Supplemental Figure 1H) and restored the survival of GPX4 cKO CD8^+^ T cells (Supplemental Figure 1, I and J). Furthermore, GPX4 deficiency significantly decreased the number (Figure 1F), proliferation (Figure 1G), and IFN-γ production (Figure 1H) of CD8^+^ T cells, and diminished completely the capability of exogenous GSH to promote the expansion and function of CD8^+^ T cells. Lip-1 addition not only recovered the GPX4 deficiency–mediated effects, but also augmented the accumulation and function of CD8^+^ T cells with addition of exogenous GSH (Figure 1, F–H). To determine the role of GPX4 in the CD8^+^ T cell–mediated antitumor response, we inoculated MC38 tumor cells in control and GPX4 cKO hosts. GPX4 deficiency in T cells significantly accelerated tumor growth (Figure 1I) and mitigated the accumulation (Figure 1J) and function (Figure 1K) of CD8^+^ T cells accompanying decreased infiltration of total CD4^+^ T cells (Supplemental Figure 1K) and CD4^+^Foxp3^+^ regulatory T cells (Supplemental Figure 1L) in comparison with the control mice. To investigate further, we used CD8-cre mice to achieve CD8^+^ T cell–specific GPX4 cKO (CD8-cre^+^ *gpx4^fl/fl^*), revealing sufficient suppression of tumor growth by host GPX4-deficient CD8^+^ T cells (Figure 1L). Furthermore, we transferred the in vitro*–*activated either control or GPX4 cKO OT-1 CD8^+^ T cells into EG7-bearing mice. Notably, GPX4 cKO CD8^+^ T cells failed to completely eradicate tumors (Figure 1, M and N) with fewer tumor-infiltrating transferred CD8^+^IFN-γ^+^ cells (Figure 1O) compared with control CD8^+^ T cells. Transferred intratumoral OT-1 GPX4 cKO CD8^+^ T cells displayed a similar exhausted-like phenotype (expressing PD-1 and Lag-3) (Supplemental Figure 1M) but an increased stem-like phenotype (expressing Ly108 and CD69) (Supplemental Figure 1N) compared with control CD8^+^ T cells. We found that PD-1^+^ non-exhausted-like T cells (PD-1^+^Lag-3^–^) displayed higher levels of GSH than exhausted-like (PD-1^+^Lag-3^+^) and other control cell subsets (Supplemental Figure 1O), while stem-like progenitors (Ly108^+^CD69^+^) displayed the highest levels of GSH among all cell subsets in transferred infiltrating CD8^+^ T cells (Supplemental Figure 1P). Likewise, GPX4 levels were higher in stem-like progenitors (Ly108^+^CD69^+^) than other cell subsets (Supplemental Figure 1Q). GPX4 levels were comparable in exhausted-like (PD-1^+^Lag-3^+^) and other activated PD-1^+^ or Lag-3^+^ cells, but lower in PD-1^–^Lag-3^–^ cells (Supplemental Figure 1R). These results indicate that GSH/GPX4 levels are likely correlated with CD8^+^ T cell stemness rather than exhaustion. Nevertheless, further investigations are needed to explore the role of GSH/GPX4 in T cell stemness and exhaustion. Consistent with the in vitro results above, transfer of Lip-1 ex vivo–treated 2C CD8^+^ T cells significantly inhibited tumor growth in B16-SIY–bearing mice (Supplemental Figure 2, A–C) accompanied by an increased accumulation and effector function of transferred CD8^+^ T cells within tumors (Supplemental Figure 2, D–H). These data suggest that GSH-mediated effects in the expansion, survival, and antitumor activity of CD8^+^ T cells are dependent primarily on GPX4.

### Modulation of CD8^+^ T cell responses by coordination of A2AR signaling and GSH metabolism.

**Figure 2:**
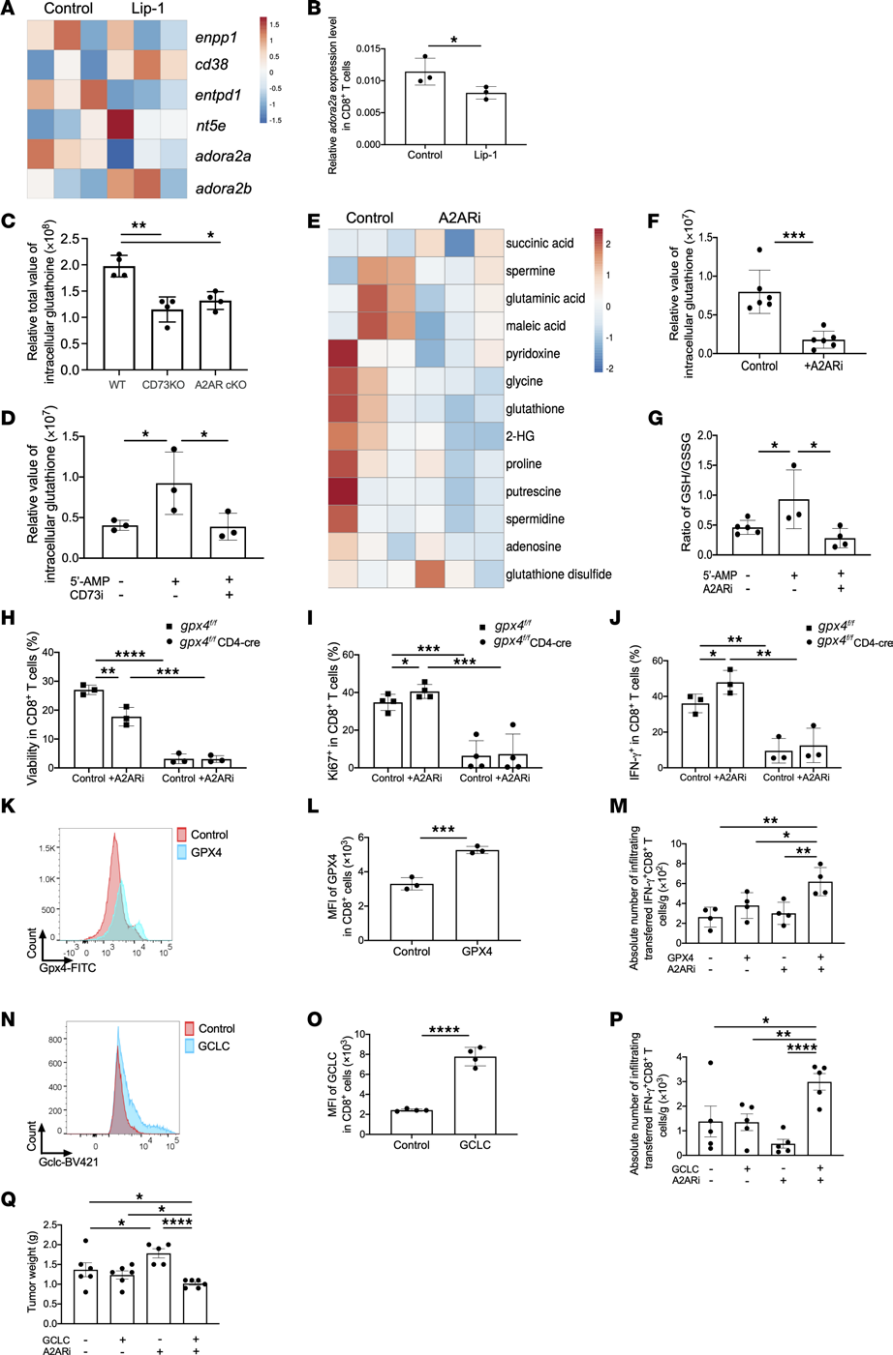
Inactivation of A2AR signaling orchestrates CD8^+^ T cell responses by coordinating the GSH/GPX4 axis. (**A**) RNA-Seq was performed in activated Pmel CD8^+^ T cells treated with or without Lip-1. Heatmap shows enriched genes involved in the adenosine pathway. (**B**) qRT-PCR analysis of *adora2a* transcripts in activated CD8^+^ T cells. (**C**) Total GSH content including both reduced (GSH) and oxidized (GSSG) forms was measured in activated CD8^+^ T cells from indicated mice in vitro with HPLC. (**D**–**G**) Relative levels of indicated metabolites were measured by LC-MS in activated Pmel CD8^+^ T cells treated as indicated (**D–F**). The GSH/GSSG ratio was calculated (**G**). The percentages and/or numbers of viable (**H**), Ki67^+^ (**I**), and IFN-γ^+^ (**J**) cells were measured in activated *gpx4^fl/fl^ CD4cre* OT-1 CD8^+^ T cells. (**K** and **L**) Flow cytometry analysis of ectopic GPX4 expression in activated WT CD8^+^ T cells. (**M**) Numbers of transferred CD8^+^IFN-γ^+^ cells were measured within MC38 tumors (*n* = 4) 14 days after i.v. transfer of activated CD90.1^+^CD8^+^ T cells with or without ectopic expression of GPX4. (**N** and **O**) Flow cytometry analysis of ectopic GCLC expression in activated OT-1 CD90.1^+^CD8^+^ T cells. (**P**) Numbers of transferred CD8^+^IFN-γ^+^ cells were analyzed within LLC1-OVA tumors (*n* = 8) 14 days after i.v. transfer of activated OT-1 CD90.1^+^CD8^+^ T cells with or without ectopic expression of GCLC. (**Q**) Tumor weights were also recorded. Results are representative of 2 (**A**, **B**, **D**–**G**, and **K**–**Q**) or 3 (**C** and **H**–**J**) independent experiments. Data were analyzed by 2-tailed *t* test (**B**, **F**, **L**, and **O**) and 2-way ANOVA (**C**, **D**, **G**, **H**–**J**, **M**, **P**, and **Q**). Data plotted are mean ± SEM from biological replicates. *P < 0.05, **P < 0.01, ***P < 0.001, ****P < 0.0001.

The CD73-mediated adenosinergic pathway suppresses CD8^+^ T cell–mediated antitumor activity (27–30). Pathway enrichment analysis based on bulk RNA-Seq data revealed that cellular response to hypoxia was among the top enrichment pathways in Lip-1–treated CD8^+^ T cells (Supplemental Figure 3A). Particularly, Lip-1 treatment decreased the abundance of *adora2a* transcript among the hypoxia-associated adenosinergic system (Figure 2A), which was also confirmed by quantitative reverse transcriptase PCR (qRT-PCR) (Figure 2B). Additionally, the pathway enrichment analysis revealed that pathways related to oxidative stress and redox were among the top enrichment pathways in response to CD73 blockade (Supplemental Figure 3B). The gene targets for GSH metabolism that promote de novo synthesis of GSH, redox homeostasis, and cellular signaling were the most enriched gene signatures (Supplemental Figure 3, C and D). Furthermore, GSH levels were measured in activated CD73-deficient and A2AR-deficient CD8^+^ T cells as compared with WT cells (Figure 2C). Either CD73 or A2AR deficiency caused a significant reduction in GSH levels in activated CD8^+^ T cells (Figure 2C). We also confirmed a decrease of extracellular adenosine accumulation from these activated CD73-deficient CD8^+^ T cells in vitro (Supplemental Figure 3E). Addition of the CD73 ectoenzymatic substrate 5′-AMP increased intracellular GSH, which was diminished by anti-CD73 treatment. This indicates that cellular GSH availability is regulated by the CD73-mediated adenosinergic pathway (Figure 2D). To address this in an unbiased way, we profiled cellular metabolites of activated CD8^+^ T cells after treatment with A2AR inhibition using a selective A2AR antagonist, SCH58261 (A2ARi), by liquid chromatography–mass spectrometry (LC-MS). Among all detected metabolites, GSH was among the top reduced metabolites in A2Ai-treated cells (Figure 2, E and F). Meanwhile, A2ARi increased levels of GSSG, leading to a decreased ratio of GSH to GSSG in the presence of 5′-AMP (Figure 2G).

Owing to the pertinent interactions of GSH metabolism and A2AR signaling, we examined how GSH affected CD8^+^ T cell responses after inactivation of A2AR signaling. A2ARi treatment (Supplemental Figure 4, A and B) increased the number of CD8^+^ T cells, and both gene deficiency and drug treatment promoted proliferation (Supplemental Figure 4, A and E) and IFN-γ production (Supplemental Figure 4, C and F) of CD8^+^ T cells. This A2AR blockade–mediated effect to enhance IFN-γ secretion was further augmented by addition of GSH but diminished completely by BSO treatment (Supplemental Figure 4, C and F). Notably, the addition of GSH enabled recovery of defects mediated by BSO (Supplemental Figure 4, A–F). We also found that GPX4 cKO CD8^+^ T cells failed to respond to A2ARi, having little capacity to affect the viability (Figure 2H), proliferation (Figure 2I), and IFN-γ production (Figure 2J) of CD8^+^ T cells compared with control cells. Similar results were observed when the GPX4 inhibitor RSL-3 was added (Supplemental Figure 4, G–J). Furthermore, Lip-1 completely restored the responses of GPX4 cKO CD8^+^ T cells and further promoted the A2ARi-mediated effects on CD8^+^ T cell control group (Supplemental Figure 4, K–M).

We further examined the effect of ectopic expression of GPX4 on CD8^+^ T cell activity upon A2ARi treatment. Increased GPX4 expression levels were confirmed in activated CD8^+^ T cells after transfection with a PiggyBac transposon system (Figure 2, K and L). CD8^+^ T cells with or without ectopic expression of GPX4 were subsequently treated ex vivo in the presence or absence of A2ARi before being transferred into MC38-bearing mice. Fourteen days after T cell transfer, the greatest numbers of IFN-γ–producing intratumoral CD8^+^ T cells (Figure 2M) were observed in mice that received A2ARi-treated GPX4-overexpressing CD8^+^ T cells, but with similar exhausted-like phenotype (expressing PD-1) (Supplemental Figure 4N) and stem-like phenotype (expressing Ly108) (Supplemental Figure 4O). Similarly to GPX4, GCLC, a key enzyme for glutamine-derived GSH biosynthesis, was ectopically expressed in OT-1 CD8^+^ T cells through a PiggyBac transposon system (Figure 2, N and O) and treated ex vivo with or without A2ARi. Furthermore, after transfer into LLC1-OVA tumor–bearing mice, the greatest numbers of IFN-γ–producing intratumoral CD8^+^ T cells were observed in mice that received A2ARi-treated GCLC-overexpressing CD8^+^ T cells (Figure 2P) accompanied by a decrease in tumor burden compared with other groups of mice (Figure 2Q). These results suggest that antitumor responses of CD8^+^ T cells are potentiated by a coordination of A2AR and the GSH/GPX4 axis.

### Inactivation of A2AR pathway reshapes activated CD8^+^ T cell metabolism toward GSSG accumulation for ferroptosis induction.

**Figure 3:**
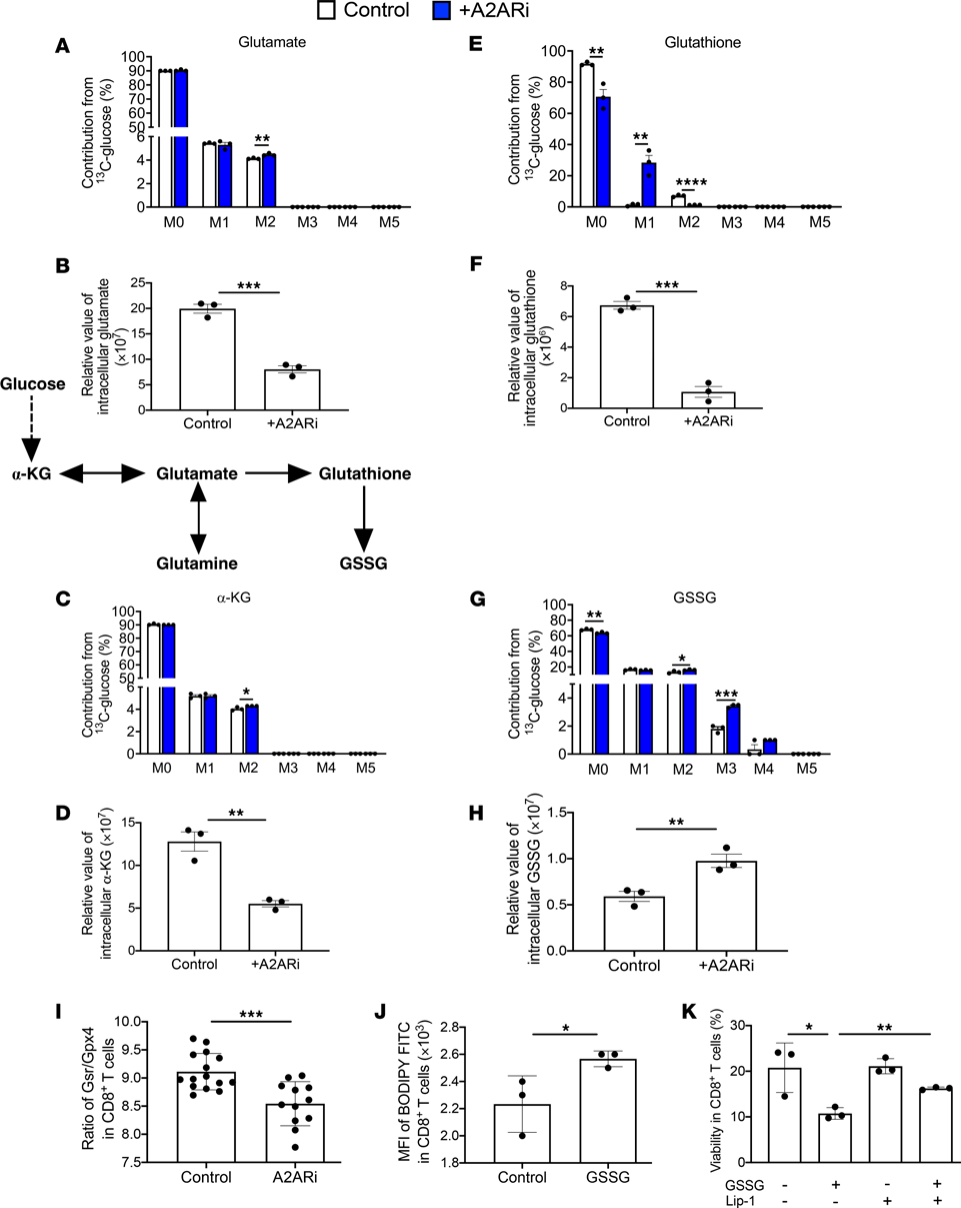
Inactivation of A2AR reshapes activated CD8^+^ T cell metabolism toward GSSG accumulation. (**A**–**H**) LC-MS analysis of incorporation of [^13^C]glucose-derived carbons into glutamate (**A**), α-KG (**C**), GSH (**E**), and GSSG (**G**), as well as relative abundance of overall glutamate (**B**), α-KG (**D**), GSH (**F**), and GSSG (**H**) among all detected metabolites involving TCA cycle and GSH synthesis after stable isotope labeling with [U-^13^C]glucose on day 7. (**I**) Ratio of GSR/GPX4 at protein level by Western blot was calculated in control and A2ARi-treated groups. (**J** and **K**) CD8^+^ T cells from WT were activated by anti-CD3 and anti-CD28 with or without cell-permeable GSSG, Lip-1, or combination. After 7 days, the BODIPY FITC signal (**J**) and percentages of viable cells (**K**) were measured by flow cytometry. Results are representative of 2 independent experiments. Data were analyzed by 2-tailed *t* test (**B**, **F**, **D**, and **H**–**J**) and 2-way ANOVA (**A**, **C**, **E**, **G**, and **K**). Data plotted are mean ± SEM from biological replicates. *P < 0.05, **P < 0.01, ***P < 0.001, ****P < 0.0001.

We next sought to determine the metabolic consequence(s) of A2ARi treatment in T cell glycolysis in the presence of GSH, considering that de novo GSH synthesis contributes to the activation-induced glycolysis in T cells (18). Although the [U-^13^C]glucose isotope-labeling assay showed slightly increased incorporation of [^13^C]glucose-derived carbons into glutamate (Figure 3A), α-ketoglutarate (α-KG) (Figure 3C), and GSH (Figure 3E) as well as GSSG (Figure 3G), the relative levels of overall glutamate (Figure 3B), α-KG (Figure 3D), and GSH (Figure 3F) among all detected metabolites decreased. However, the relative levels of GSSG significantly increased (Figure 3H), indicating that glucose may serve as a fuel source for ultimate GSSG accumulation in activated CD8^+^ T cells through rapid oxidization of GSH and utilization of its precursor amino acid, glutamate, upon A2ARi treatment, while decreasing a cellular need for the TCA cycle. Meanwhile, the ratio of GSR to GPX4 at the protein level was decreased in the A2ARi-treated group compared with the control group (Figure 3I), indicating that the GSSG accumulation upon A2ARi treatment is caused likely by an impaired capacity to recycle GSSG back to GSH. We then showed that A2ARi treatment caused more cellular death in activated CD8^+^ T cells with addition of exogenous GSH, and Lip-1 treatment restored the viability of T cells (Supplemental Figure 5, A and B). We also observed increased lipid ROS levels in CD8^+^ T cells after A2ARi treatment with addition of exogenous GSH (Supplemental Figure 5, C and D), consistent with the imbalance of the ratio of GSH to GSSG during oxidative stress (Figure 2G). In addition, activation of A2AR signaling using the A2AR-selective agonist CGS 21680 increased T cell survival with addition of exogenous GSH (Supplemental Figure 5, E and F). These data suggest that activated CD8^+^ T cells preferentially undergo ferroptosis upon A2AR signaling inactivation in the GSH-rich environment. We also found that the frequency of CD39^+^CD73^+^ cells was increased significantly in both naive and activated GPX4-deficient CD8^+^ T cells (Supplemental Figure 5G), confirming further the potential relevance of adenosine signaling to T cell ferroptosis induced by GPX4 ablation. Given that GSSG was significantly accumulated in the A2ARi-treated activated CD8^+^ T cells (Figure 3H), we hypothesized that the T cell ferroptosis induced by A2ARi could be due to the enhanced GSH usage and subsequent increased GSSG accumulation. To test this hypothesis further, we added the cell-permeable GSSG in CD8^+^ T cell cultures. As expected, GSSG led to an increase in lipid ROS (Figure 3J) accompanied by a decrease in T cell viability (Figure 3K). Notably, addition of Lip-1 abrogated the GSSG-mediated cellular death (Figure 3K). Collectively, our data indicate that the balance of the ratio of GSH to GSSG is fine-tuned by A2AR signaling to maintain T cell survival and function.

Our RNA sequence on activated CD8^+^ T cells from control and A2ARi treatment also confirms that A2ARi treatment affects GSH metabolism. Transcriptome analysis of pathway enrichment (Supplemental Figure 6A) and gene set enrichment analysis (GSEA) Supplemental Figure 6B) indicated that the hallmark pathway of glutamine family amino acid metabolic process was among the top-ranking upregulated gene sets in A2ARi-treated cells. A2ARi treatment caused enhanced expression of *gls*, *gls2*, *gclm*, *gclc*, and *slc7a11* (which encode the key enzymes and transporter for glutamine-derived GSH biosynthesis) as well as *slc25a39* (which encodes the only mitochondrial membrane transporter for mitochondrial GSH import), driving the de novo GSH biosynthesis and the mitochondrial GSH-import machinery, respectively (Supplemental Figure 6C). We then sought to explore the involvement of A2AR-mediated downstream signal pathways such as protein kinase A (PKA) in modulation of GSH metabolism. The RNA-Seq (Supplemental Figure 6D) and immunoblotting data (Supplemental Figure 6, E and F) confirmed a positive correlation of expression levels between A2AR and PKA. We then added the PKA pathway inhibitor H89 to mimic the effect of A2ARi in compromising activated T cell survival (Supplemental Figure 6, G and H) while upregulating GSH metabolic genes (*gclc*, *gclm*, and *gpx4*) (Supplemental Figure 6I). These results are in line with the increased utilization of GSH metabolism by A2ARi (Figure 3, A–E).

### Inhibition of lipid peroxidation improves adoptive T cell therapy with A2ARi.

**Figure 4:**
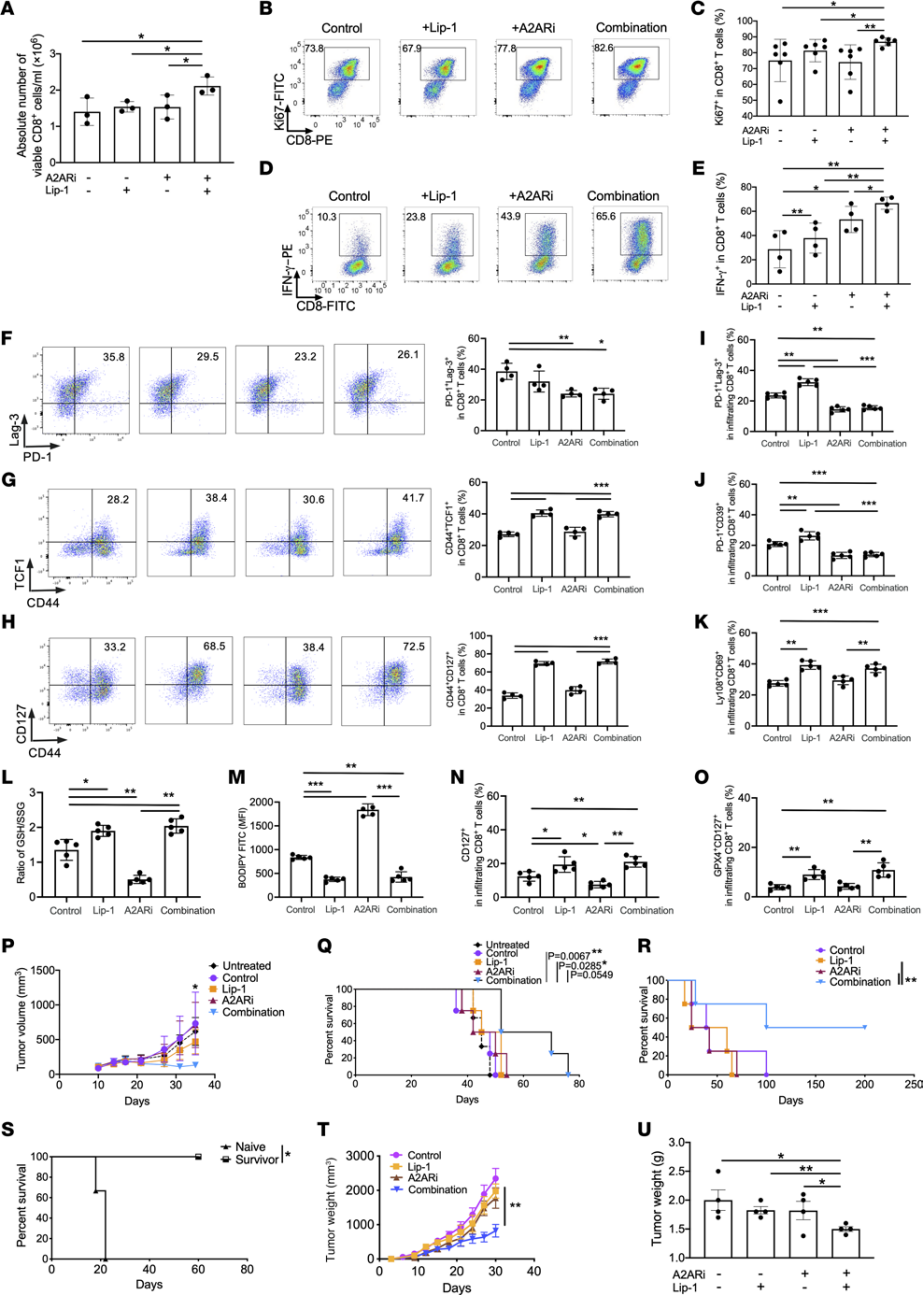
Adoptive T cell therapy with A2ARi plus Lip-1. The percentages and/or numbers of viable (**A**), Ki67^+^ (**B** and **C**), and IFN-γ^+^ (**D** and **E**) cells were measured in activated CD8^+^ T cells as indicated. The percentages of viable PD-1^+^Lag-3^+^ (**F**), CD44^+^TCF1^+^ (**G**), and CD44^+^CD127^+^ (**H**) cells were measured in activated CD8^+^ T cells as indicated. Percentages of PD-1^+^Lag-3^+^ (**I**), PD-1^+^CD39^+^ (**J**), Ly108^+^CD69^+^ (**K**), CD127^+^ (**N**), or CD127^+^GPX4^+^ (**O**) cells among transferred cells in EG7 tumor infiltrates (*n* = 5) were measured 1 week after i.v. transfer of activated OT-1 CD90.1^+^CD8^+^ T cells. In parallel, infiltrating transferred T cells were sorted and measured for intracellular GSH and GSSG by HPLC. The GSH/GSSG ratio was calculated (**L**). Lipid ROS levels indicated by BODIPY FITC were also detected in transferred infiltrating T cells by flow cytometry (**M**). Tumor volume (**P**) and survival (**Q**) were measured in MC38-bearing mice (*n* = 5) with and without i.v. transfer of activated tumor-reactive CD90.1^+^CD8^+^ T cells as indicated. (**R** and **S**) EG7-bearing mice (*n* = 5) received i.v. transfer of activated OT-1 CD90.1^+^CD8^+^ T cells as indicated. Mouse survival was measured until day 200 (**R**). Tumor-free mice from **R** were rechallenged with EG7 cells, while naive mice received the same EG7 cells simultaneously s.c., and mouse survival was measured over time (**S**). Tumor volume (**T**) and weight (**U**) (on day 28) were measured in HCC-1806–bearing mice (*n* = 4) with activated anti-MSLN CAR T cells as indicated. Results are representative of 2 (**F**–**H** and **P**–**S**) or 3 (**A**–**E**, **T**, and **U**) independent experiments. Data were analyzed by 2-way ANOVA (**A**, **C**, **E**, **F**–**O**, **T**, and **U**) and log-rank test for survival curves (**Q** and **R**). Data plotted are mean ± SEM from biological replicates. *P < 0.05, **P < 0.01, ***P < 0.001.

As A2ARi induced better effector function of CD8^+^ T cells along with more ferroptosis, we then combined A2ARi and Lip-1 to test whether combination therapy would induce superior responses in CD8^+^ T cells. As expected, our combination therapy promoted T cell expansion (Figure 4, A–C) and IFN-γ production (Figure 4, D and E) in vitro and enhanced polyfunctionality (Supplemental Figure 7A) and polyfunctional strength index (Supplemental Figure 7B) of activated CD8^+^ T cells determined by IsoPlexis single-cell functional proteomics in vitro as compared with single treatment alone. Treatment with A2ARi or with A2ARi plus Lip-1 resulted in a significant reduction of exhausted-like phenotype in activated OT-1 CD8^+^ T cells in the cultures (Figure 4F), while the stem-like phenotype (expressing TCF1 and CD44) (Figure 4G) and memory phenotype (expressing CD127 [IL-7Rα] and CD44) (Figure 4H) were increased upon treatment with Lip-1 or with Lip-1 plus A2ARi in comparison with control CD8^+^ T cells. Consistent with these in vitro results, following adoptive transfer these OT-1 T cells treated with A2ARi or A2ARi plus Lip-1 displayed reduced exhausted-like phenotype within EG7 tumors, although Lip-1 treatment increased T cell exhausted-like phenotype (Figure 4, I and J). Likewise, treatment with Lip-1 or with Lip-1 plus A2ARi increased stem-like phenotype (Figure 4K) of transferred OT-1 T cells, accompanied by an increase in the ratio of GSH to GSSG (Figure 4L) but a decrease in the levels of lipid ROS (Figure 4M) in comparison with control or A2ARi-treated CD8^+^ T cells. In addition, in line with the previously established observations with respect to the role of A2AR signaling in T cell persistence and quiescence state by suppression of TCR-induced activation involving IL-7Rα–mediated signaling (31, 32), transferred tumor-infiltrating OT-1 T cells decreased CD127 expression upon A2ARi treatment. However, treatment with Lip-1 or with Lip-1 plus A2ARi increased CD127 expression on transferred tumor-infiltrating OT-1 T cells (Figure 4N). Moreover, the CD127 expression was correlated with the levels of GPX4 in these infiltrating T cells (Figure 4O). Establishment of the relationship between GPX4/A2AR and IL-7Rα–mediated signaling in naive and tumor antigen–experienced T cells warrants further investigation. We also transferred the in vitro–treated purified WT polyclonal MC38-reactive CD8^+^ T cells in MC38 tumor–bearing hosts. Consistently, transfer of activated CD8^+^ T cells with A2ARi plus Lip-1 resulted in a greater inhibition of tumor growth (Figure 4P) and a longer overall survival (Figure 4Q) of hosts than those with each treatment alone. Similar antitumor effects were obtained when the in vitro–treated purified OT-1 CD8^+^ T cells were transferred into EG7 tumor–bearing hosts (Figure 4R). Within the group that received T cells with combination treatment, 50% of the animals remained tumor free until 200 days (Figure 4R). We next tested whether mice cured with this combined therapy developed long-term immune memory. We rechallenged the mice that had rejected the EG7 tumor with the same EG7 cells compared with naive mice injected with EG7 cells simultaneously. These rechallenged mice rejected the implanted EG7 cells with a long-term survival, while tumor growth was aggressive in naive mice (Figure 4S). We next examined the effects of combination treatment on human anti-MSLN chimeric antigen receptor T (CAR-T) cells. Notably, combination treatment induced a higher polyfunctionality of CAR-T cells with increased cytokine composition of co-secreting IFN-γ, granzyme B, and perforin, etc., at single-cell level in vitro compared with each treatment (Supplemental Figure 7, C and D) and enabled improvement of antitumor activity of CAR-T cells in NOD *scid* gamma (NSG) mice bearing HCC-1806 tumors (Figure 4, T and U). Additionally, the qPCR data revealed that A2ARi significantly increased gene expression of *gpx4* and *gclc*, and the addition of Lip-1 further resulted in increased expression of these GSH-related genes in the presence of A2ARi (Supplemental Figure 8, A and B). These results suggest a synergistic effect of modulation of both GSH metabolism and A2AR signaling. Combination of inhibition of lipid peroxidization and inactivation of A2AR can prevent ferroptosis and facilitate T cell effector function through the GSH pathway, leading to a persistent antitumor response of CD8^+^ T cells. On the other hand, activated CD8^+^ T cells predominantly expressed *gpx1* and *gpx4* rather than other isoforms of GPXs (Supplemental Figure 8C). Despite adenosine-dependent induction of *gpx1* in human primary endothelial cells and cellular protection against oxidative stress (33), we found that treatment with A2ARi or the A2AR agonist CGS 21680 altered the gene expression of *gpx4* but not *gpx1* (Supplemental Figure 8D) determined by RT-qPCR, indicating the importance of A2AR-mediated signaling in specific regulation of *gpx4* transcripts in activated CD8^+^ T cells. A2ARi treatment also upregulated gene levels of other antioxidant enzymes, *txnrd1*, *prdx1*, *prdx3*, and *park7* (Supplemental Figure 8E), though their expression levels were lower than that of *gpx4* (Supplemental Figure 8F). Taken together, these results suggest that the proposed mechanism involving A2AR signaling is dependent largely on *gpx4* rather than other isoforms of GPXs in activated CD8^+^ T cells, while the potential contribution of other antioxidant enzymes requires further investigation.

**Figure 5:**
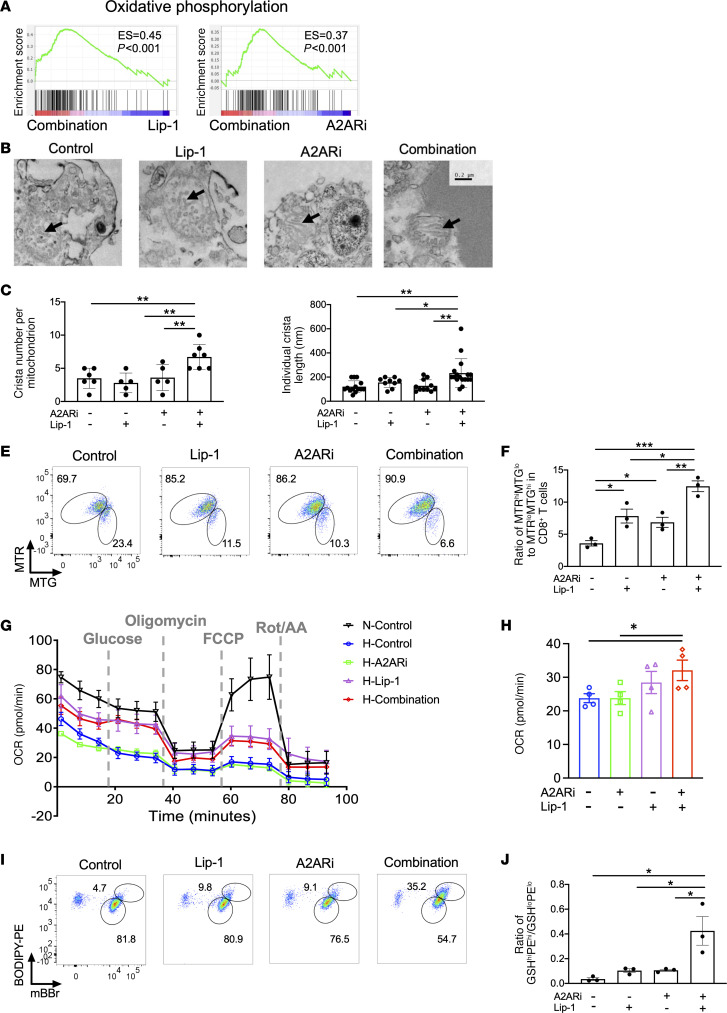
Combination of Lip-1 and A2ARi promotes mitochondrial function in activated CD8^+^ T cells. (**A**–**D**) Activated OT-1 CD8^+^ T cells in the presence of OVA-I, anti-CD28, and GSH were treated with or without Lip-1, A2ARi, or a combination of both. RNA-Seq was performed in activated T cells, and GSEA plot showed the enriched pathway of oxidative phosphorylation in the combination of both compared with single treatment alone (**A**). Activated CD8^+^ T cells were fixed and observed by transmission electron microscopy (**B**). The number (**C**) and length (**D**) of cristae in mitochondria were measured and summarized. (**E** and **F**) Activated CD8^+^ T cells were stained with MTR and MTG to measure the mitochondrial membrane potential and mitochondrial mass, respectively (**E**). The ratio of MTR^hi^MTG^lo^ to MTR^lo^MTG^hi^ was calculated (**F**). (**G** and **H**) The oxygen consumption rate (OCR) of activated OT-1 CD8^+^ T cells treated as indicated in the presence of OVA-I, anti-CD28, and GSH under normoxic (N) or hypoxic (H) conditions was measured by the Seahorse MitoStress Test with injections of oligomycin, FCCP, and antimycin A/rotenone (**G**). Basal OCR (**H**) was calculated from these activated CD8^+^ T cells cultured under hypoxic conditions. (**I** and **J**) Activated CD8^+^ T cells were stained with BODIPY and mBBr to measure neutral lipid by PE signal and GSH, respectively (**I**). The ratio of PE^hi^GSH^hi^ to PE^lo^GSH^lo^ was calculated (**J**). Results are representative of 2 (**A**–**D**) or 3 (**E**, **F**, **I**, and **J**) independent experiments. Data were analyzed by 2-way ANOVA (**C**, **D**, **F**, **H**, and **J**). Data plotted are mean ± SEM from biological replicates. *P < 0.05, **P < 0.01, ***P < 0.001.

We next sought to address how the combination treatment affected activated CD8^+^ T cells. The GSEA analysis from RNA-Seq showed that oxidative phosphorylation was among the top overlapped enriched pathways (Supplemental Figure 8C) in activated CD8^+^ T cells with combination treatment as compared with those with single treatment alone (Figure 5A). The imbalance of the ratio of GSH to GSSG during oxidative stress could contribute to mitochondrial dysfunction in T cells. To address this, we examined mitochondrial structures and numbers by electron microscopy (Figure 5B). Notably, the crista number (Figure 5C) and length per mitochondrion (Figure 5D) in activated CD8^+^ T cells were elevated after combination treatment. We also measured mitochondrial mass and membrane potential in activated CD8^+^ T cells by staining with MitoTracker Green (MTG) and MitoTracker Deep Red (MTR) (Thermo Fisher Scientific), respectively (Figure 5E). The ratio of MTR to MTG, an indicator of normalized mitochondrial activity, was increased in treated T cells upon A2ARi or Lip-1 alone (Figure 5F). This effect was further enhanced by a combination of both (Figure 5F). Consistently, A2AR pathway–induced activation of PKA signaling was also required for regulation of lipid ROS (Supplemental Figure 8H) and potential mitochondrial function (Supplemental Figure 8I) in activated CD8^+^ T cells. In addition, activated CD8^+^ T cells cultured under hypoxia conditions exhibited a significantly lower basal oxygen consumption rate (OCR) than those cultured under normoxic conditions (Figure 5G). Notably, compared with A2ARi or Lip-1 treatment alone, combination of both increased the OCR in activated CD8^+^ T cells cultured under hypoxia conditions (Figure 5H). These data together support that inactivation of A2AR plus inhibition of lipid peroxidization maintains superior CD8^+^ T cell responses coinciding with improved utilization of mitochondrial activity. Oxidation of the polyunsaturated butadienyl portion of the BODIPY 581/591 dye results in a shift of the fluorescence emission peak from –590 nm (red, phycoerythrin [PE] channel) to –510 nm (green, FITC channel). Combining this with mBBr staining that measures intracellular GSH content (Figure 5I), the ratio of BODIPY-PE^hi^mBBr^hi^ to BODIPY-PE^lo^mBBr^lo^ could thus be potentially indicative of the capacity of activated cells to maintain resistance to ferroptosis. Indeed, the ratio of BODIPY-PE^hi^mBBr^hi^ to BODIPY-PE^lo^mBBr^lo^ was significantly increased in the activated CD8^+^ T cells with treatment of A2ARi plus Lip-1 compared with A2ARi or Lip-1 treatment alone (Figure 5J).

### Transcriptional regulation of gpx4 by E2F1.

**Figure 6:**
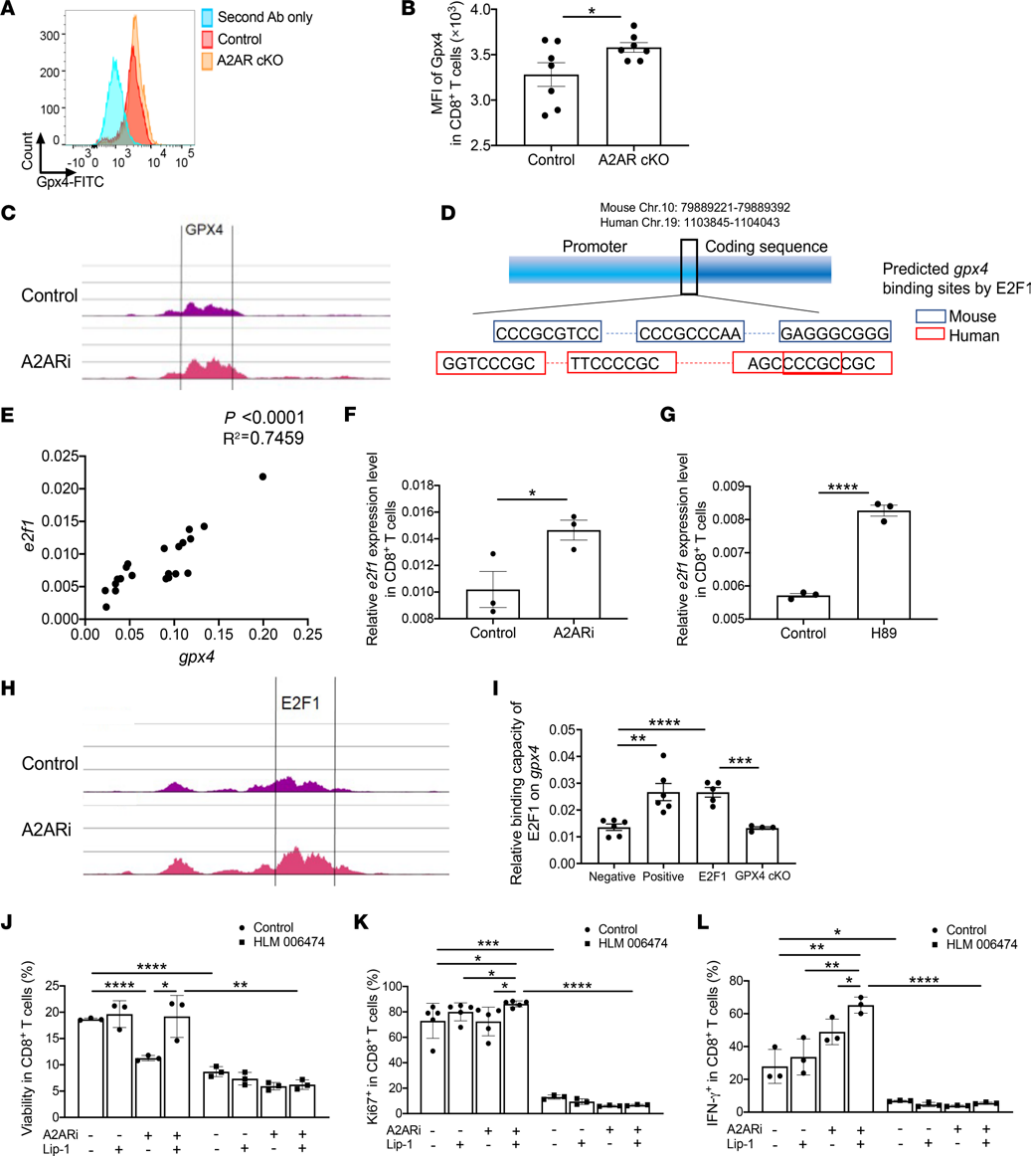
Transcriptional regulation of *gpx4* by E2F1. (**A** and **B**) GPX4 expression level was evaluated by flow cytometry in activated WT and *adora2a^fl/fl^ Lck^cre^* cKO CD8^+^ T cells. (**C**) ATAC-seq analysis by Partek Flow software showing chromatin accessibility within the vicinity of transcribed regions for GPX4 in activated Pmel CD8^+^ T cells treated with or without A2ARi. (**D**) Predicted binding sites by PROMO-ALGGEN Web tool for human and mouse *gpx4* by E2F1. (**E**) Correlation between gene expression levels of *e2f1* and *gpx4* by qRT-PCR in activated Pmel CD8^+^ T cells. (**F** and **G**) qRT-PCR was performed to detect expression levels of *e2f1* in activated Pmel CD8^+^ T cells treated with or without A2ARi (**F**) or with or without H89 (**G**). (**H**) ATAC-seq analysis by Partek Flow showing chromatin accessibility within the vicinity of transcribed regions for E2F1 in activated Pmel CD8^+^ T cells treated with or without A2ARi. (**I**) ChIP-qPCR analysis confirming the binding capacity of E2F1 on *gpx4* in activated control OT-1 CD8^+^ T cells rather than *gpx4^fl/fl^ CD4^cre^* OT-1 CD8^+^ T cells. (**J**–**L**) Activated Pmel CD8^+^ T cells in the presence of gp100, anti-CD28, 5′-AMP, and cell-permeable GSH were treated with or without A2ARi, Lip-1, E2F1 inhibitor HLM 006474, or combination. On day 7, the percentages of viable (**J**), Ki67^+^ (**K**), and IFN-γ^+^ (**L**) T cells were measured. Results are representative of 2 (**C**, **H**, and **I**) or 3 (**B**, **E**–**G**, and **J**–**L**) independent experiments. Data were analyzed by Pearson’s correlation (**E**), 2-tailed *t* test (**B**, **F**, and **G**), and 2-way ANOVA (**I**–**L**). Data plotted are mean ± SEM from biological replicates. *P < 0.05, **P < 0.01, ***P < 0.001, ****P < 0.0001.

Given the interaction between the GSH/GPX4 axis and A2AR signaling in CD8^+^ T cells, we next investigated the molecular mechanism underlying regulation of GPX4 by A2AR signaling in activated CD8^+^ T cells. We found that A2AR deficiency in CD8^+^ T cells upregulated GPX4 expression (Figure 6, A and B) and A2ARi upregulated gene expression of *gpx4*, which was supported by assay for transposase-accessible chromatin using sequencing (ATAC-seq) data showing increased chromatin accessibility within the vicinity of transcribed regions for *gpx4* in activated CD8^+^ T cells upon A2ARi treatment (Figure 6C), pointing to a possible transcriptional regulation of GPX4 expression by inactivation of A2AR signaling. A number of transcription factors (TFs) shared by humans and mice that are predicted to bind *gpx4* were identified through the PROMO-ALGGEN Web tool (https://alggen.lsi.upc.es/cgi-bin/promo_v3/promo/promoinit.cgi?dirDB=TF_8.3) (Figure 6D). Among those TFs, *e2f1* showed a robust positive correlation with the gene expression level of *gpx4* in CD8^+^ T cells (Figure 6E). We found expression of *e2f1* upregulated similarly to that of *gpx4* upon A2ARi or H89 treatment by qPCR analysis (Figure 6, F and G). Consistently, ATAC-seq analysis confirmed a correlation between *e2f1* and *gpx4* in chromatin accessibility, particularly in response to A2ARi (Figure 6H). Moreover, chromatin immunoprecipitation (ChIP)–qPCR analysis further validated the binding capacity of E2F1 on *gpx4* in control activated CD8^+^ T cells rather than GPX4 cKO cells (Figure 6I). We next tested the functional consequences of E2F1 inhibition for T cells using an E2F1 inhibitor, HLM 006474. Notably, similar to the effects mediated by GPX4 deficiency, E2F1 inhibition decreased the survival (Figure 6J) and function (Figure 6, K and L) of activated CD8^+^ T cells and completely abolished the activities induced by A2ARi, Lip-1, or a combination of both compared with the control settings without E2F1 inhibition. These data suggest a pivotal role of E2F1-mediated transcriptional modulation of *gpx4* expression involving A2AR signaling in sustaining optimal CD8^+^ T cell responses.

### Identification of the GSH metabolism–related gene signature (GMGS) in intratumoral CD8^+^ T cells across a range of human cancers.

**Figure 7:**
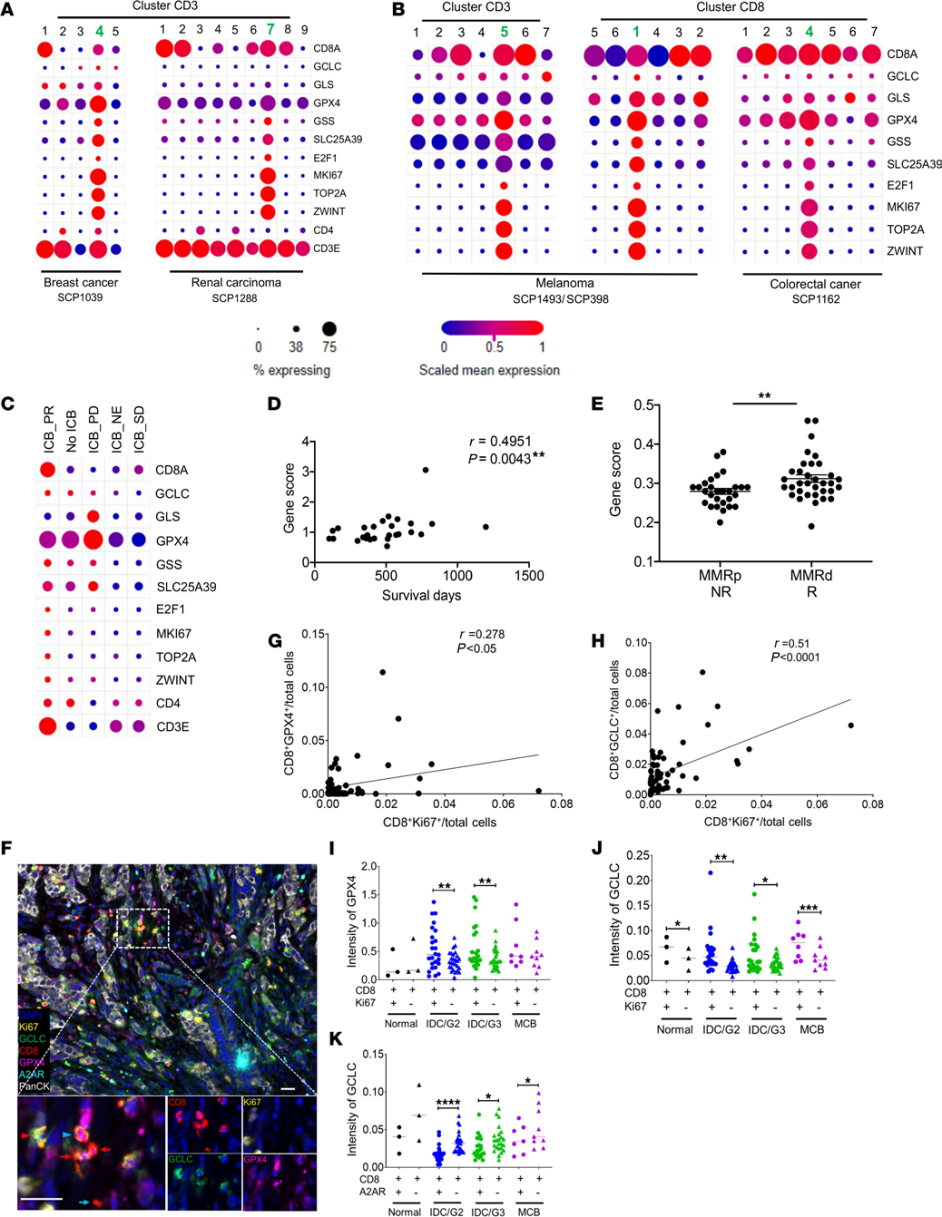
Identification of a tumor-infiltrating CD8^+^ T cell cluster expressing the GSH metabolism signature genes (GMSG) across a range of human cancers. (**A** and **B**) Dot plot of scRNA-Seq data (https://singlecell.broadinstitute.org/single_cell) showing geometric mean expression (log[TP10K+1]) and frequency (dot size) of key genes associated with GSH metabolism in clusters of tumor-infiltrating CD3^+^ and/or CD8^+^ T cells from patients with breast cancer (*n* = 26), renal carcinoma (*n* = 8), melanoma (*n* = 59), and colon cancer (*n* = 62). Clusters enriched in GSH metabolism signature genes (GMSG) are indicated in bold green. (**C**) The GMSG-enriched tumor-infiltrating CD8^+^ T cell cluster was found only in ICB_PR renal carcinoma patients (SCP1288; *n* = 2). (**D**) Correlation between the score (normalized gene expression relative to the total of tumor-infiltrating CD8^+^ T cells for a curated list of GMSG) and overall survival of melanoma patients (SCP398; *n* = 27). (**E**) Scores of GMSG in MMRd (R) (*n* = 34) and MMRp (NR) (*n* = 28) colorectal cancer patients (SCP1162). (**F**) Representative composite image of a breast cancer specimen stained by multicolor IHC comprising CD8, Ki67, GCLC, GPX4, A2AR, PanCK, and DAPI. Scale bars: 10 μm. Original magnification, ×20. Representative CD8^+^GPX4^+^ (blue arrow), CD8^+^GCLC^+^ (blue arrowhead), CD8^+^GPX4^+^GCLC^+^ (red arrows), and CD8^+^Ki67^+^GPX4^+^GCLC^+^ (red arrowhead) cells. (**G** and **H**) Correlation between CD8^+^Ki67^+^ density and CD8^+^GPX4^+^ density (**G**) or CD8^+^GCLC^+^ density (**H**). (**I** and **J**) Intensity of GPX4 (**I**) or GCLC (**J**) was compared between CD8^+^Ki67^+^ cells and CD8^+^Ki67^–^ cells among cases of invasive ductal carcinoma (IDC: G2, *n* = 27; G3, *n* = 24) and medullary carcinoma of the breast (MCB, *n* = 9) and normal breast tissues (*n* = 3). (**K**) Intensity of GCLC was compared between CD8^+^A2AR^+^ cells and CD8^+^A2AR^–^ cells among cases of IDC (G2, *n* = 27; G3, *n* = 24) and MCB (*n* = 9) and normal breast tissues (*n* = 3). Data were analyzed by Pearson’s correlation (**D**, **G**, and **H**), 2-way ANOVA (**I**–**K**), and 2-sided Mann-Whitney test (**E**). *P < 0.05, **P < 0.01, ***P < 0.001, ****P < 0.0001.

Our data revealed the importance of GSH metabolism involving GSH synthesis and GPX4 for antitumor responses of CD8^+^ T cells. Furthermore, to determine the clinical significance of GSH metabolism by CD8^+^ T cells, we analyzed the single-cell sequencing data from the Single Cell Portal (Broad Institute). In 2 separate data sets on breast cancer and renal carcinoma, respectively, the GSH metabolism–related gene set, including *gclc*, *gls*, *gpx4*, *gss*, *slc25a39*, and *e2f1*, was enriched along with *mki67*, *top2a*, and *zwint* genes in one particular cluster of intratumoral CD8^+^ T cells (Figure 7A), presumably linking with a superior proliferative potential. We confirmed this observation in more independent data sets on melanoma and colorectal cancer (Figure 7B). We then established a putative gene signature (comprising *gclc*, *gls*, *gpx4*, *gss*, *slc25a39*, and *e2f1*) of GSH metabolism (GMGS) in association with therapeutic response. In renal carcinoma, the partial response (PR) patients after immune checkpoint blockade (ICB) showed preferential enrichment of GSH signature with the highest expression levels of GMGS (Figure 7C). In melanoma, the core of GMGS was positively correlated with survival days (Figure 7D). In colorectal cancer, mismatch repair–deficient (MMRd) tumors displayed increased antitumor immunity as compared with mismatch repair–proficient (MMRp) tumors (34). In line with this notion, the score of GMGS was significantly higher in MMRd patients, possibly indicating favorable antitumor immune responses (Figure 7E). Interestingly, exhausted-like gene sets (*pdcd1*, *lag3*, *tox*, *tigit*, *ctla4*, *prdm1*, and *havcr7*) but not stem-like gene sets (*tcf7* and *ccr7*) were also enriched in the CD8^+^ cluster with GMGS (Supplemental Figure 9). The results suggest that GMGS may help define a unique proliferative population of intratumoral CD8^+^ T cells distinct from conventional exhausted-like or stem-like T cells, potentially associating with therapeutic response of immunotherapy.

We next adopted an unbiased approach to investigate the presence of CD8^+^ T cells expressing GPX4 and/or GCLC, correlated with Ki67 and A2AR expression at the protein level through multiplex immunohistochemistry. The density of CD8^+^ T cells in the total analyzed area including total, tumor nest, and/or stromal compartment was significantly higher in medullary carcinoma of the breast (MCB) than in invasive ductal carcinoma (IDC), while IDC displayed a lower CD8^+^ T cell density than normal breast tissues, consistent with the hypothesis that MCB has a favorable prognosis with superior CD8^+^ T cell infiltration (35). Although there were no significant differences in intensity of GPX4 in CD8^+^ T cells in individual compartments between MCB and IDC, we found a significantly higher intensity of GCLC in CD8^+^ T cells in MCB than in IDC, particularly in each compartment (Supplemental Figure 10). Tissue sections from breast carcinoma (Figure 7F) showed distinct populations of CD8^+^GPX4^+^, CD8^+^GCLC^+^, CD8^+^GPX4^+^GCLC^+^, and CD8^+^Ki67^+^GPX4^+^GCLC^+^ cells. There was a positive correlation of CD8^+^GPX4^+^ (Figure 7G) or CD8^+^GCLC^+^ (Figure 7H) with CD8^+^Ki67^+^. Moreover, there was a higher intensity of GPX4 in CD8^+^Ki67^+^ than in CD8^+^Ki67^–^ in the case of both IDC-G2 and -G3 tumors (Figure 7I). The intensity of GCLC in CD8^+^Ki67^+^ was also higher than that in CD8^+^Ki67^–^ in the IDC and MCB tumors as well as normal breast tissues (Figure 7J). In addition, there was a lower intensity of GCLC in CD8^+^A2AR^+^ than in CD8^+^A2AR^–^ in the IDC and MCB tumors (Figure 7K). In line with our results from our mouse models, these data from clinical tumor samples further support the importance of fine-tuning the redox state in concert with inactivation of A2AR signaling in intratumoral CD8^+^ T cells for favoring the antitumor immune response. We propose a mechanism that underlies an antitumor response of CD8^+^ T cells by modulating GSH metabolism and A2AR signaling (Supplemental Figure 11).

## Discussion

Recent studies have begun to explore the roles of GSH in T cell activation and metabolic reprogramming (18, 19). The GSH system is well recognized as a major cellular antioxidant that maintains redox homeostasis in T cells (36). However, whether and how the functional fitness of activated T cells during chronic stimulation can be shaped by intracellular metabolic networks of GSH in maintaining their antitumor activity remains largely unknown. In this study, we have demonstrated that the GSH/GPX4 axis is required for antitumor responses of activated CD8^+^ T cells in driving their metabolic adaptation and mitochondrial maintenance. Mechanistically, the CD73/A2AR signaling cascade coordinates the de novo synthesis and consumption of GSH with GSSG accumulation and concomitant mitochondrial lipid peroxidation, which links potentially to PKA/E2F1–dependent fine-tuning of the ratio of GSR to GPX4. Additionally, we identify a particular cluster of intratumoral CD8^+^ T cells expressing a putative gene signature of GSH metabolism in association with clinical responses and survival across several human cancers.

GSH plays important roles in many cellular activities, including ROS removal, DNA and protein synthesis, and signal transduction (37). Besides directly buffering ROS, GSH takes part in the regeneration of the GPXs that detoxify lipid hydroperoxides and H_2_O_2_ (38). Notably, we have revealed here that sustained intracellular GSH levels are essential for CD8^+^ T cell expansion, survival, and effector function and defined the complete dependence of such regulation on GPX4 expression and activity, through a metabolic signaling axis that ties the A2AR/PKA/E2F1 pathway to GSH metabolism and lipid peroxidation.

Activated T cells without GPX4 accumulate cellular lipid peroxides and undergo ferroptosis (21, 39, 40), while ferroptotic oxidative signals are produced primarily by iron-mediated Fenton reaction or enzymatic reaction via lipoxygenases or the dysregulated GSH antioxidant system (41, 42). Consistently, emerging studies indicated that modulation of oxidative stress, ablation of CD36 (a scavenger receptor known for its function as a fatty acid transporter) (43, 44), or overexpression of GPX4 (44, 45) suppressed lipid peroxidation, restored tumor-infiltrating CD8^+^ effector functions, and enabled tumor control. While the impact of lipid uptake on GSH levels remains unclear, our data strongly argue that an essential role of GSH/GPX4 in activated CD8^+^ T cells is to sustain their effector function and protect them from ferroptosis as opposed to redox buffering. In particular, we show that inactivation of A2AR signaling reshapes the GSH metabolism in activated CD8^+^ T cells by augmenting the expression of a number of key genes involved in the de no synthesis and utilization of intracellular GSH and preferentially facilitating the GSH consumption toward GSSG. Consequently, A2AR blockade enhances T cell effector function while inducing ferroptosis due to increased lipid peroxidation, which provides a mechanistic explanation for the recent observations of decreased survival of tumor-infiltrating A2AR-deficient T cells (46) or activated CD73-deficient CD8^+^ T cells under antigenic stimulation (47). In addition, Zou’s group reported the induction of tumor cell ferroptosis due to the suppression of the GSH/GPX4 axis by CD8^+^ T cell–mediated IFN-γ upon ICB (48), confirming a generalizable ferroptotic pathway also used by tumor cells for survival (6).

Activation of aerobic glycolysis supports both proliferation and effector function of T cells upon costimulation of the TCR and CD28 receptors (49, 50). Surprisingly, however, our isotope tracing analysis shows a decreased cellular need of glucose for TCA cycle activity but increased accumulation of GSSG by rapid oxidization of GSH while maintaining T cell proliferation and effector function upon A2ARi. More importantly, inhibition of lipid peroxidation by Lip-1 not only increases proliferation and effector function (similarly to A2ARi) but also subverts A2Ai-induced ferroptosis in the activated T cells independent of GPX4, confirming a central role of lipid peroxidation in the process. Accordingly, combination treatment of A2ARi and Lip-1 improves the utilization of mitochondrial activity. When adoptively transferred, these activated CD8^+^ T cells treated with A2ARi plus Lip-1 or overexpressing GPX4/GCLC ex vivo have a superior capacity to proliferate and persist in vivo, with enhanced antitumor efficacy.

Interestingly, we found that the tumor-infiltrating CD8^+^ T cells displayed significantly higher levels of intracellular GSH and, to a greater degree, of lipid ROS than did splenic T cells. Moreover, the GPX4/GSH levels seem to be correlated with T cell stemness. Given the fact that many tumor-infiltrating CD8^+^ T cells are already anergic/exhausted, an alternative hypothesis is that the levels of GSH in the T cells are potentially more a consequence of the redox state of the hypoxic/reduced tumor microenvironment (TME) than a sign of T cell fitness, which remains to be tested. An in-depth assessment of the role of GSH/GPX4 in T cell fitness within the TME requires further investigation.

Collectively, our work demonstrates a viable strategy of A2ARi in combination with inhibition of lipid peroxidation to improve persistence and functionality of ACT against cancer, contributing to the debate over the best way to target GPX4 for cancer therapy. Emerging studies (51, 52) indicate the importance of eliminating the upstream stage of the adenosinergic axis by targeting tumor hypoxia and redox state using antihypoxic oxygenation strategies to reinvigorate immunotherapy. It is thus expected that use of oxygenation agents together with A2ARi may enable more effective ACT when combined with lipid peroxidation inhibition. In addition, further understanding of the metabolic process of GSH synthesis and modulation of GPX4 and lipid peroxidation in activated T cells relative to other cells in the TME is warranted to suggest avenues to augment current immunotherapies. Moreover, validation of GMGS-based biomarkers to predict response to immunotherapy across large cohorts of individuals may be considered in the future.

## Methods

### Sex as a biological variable.

Both male and female animals were examined in this study, and similar findings were reported for both sexes.

### Tumor challenge and adoptive T cell transfer.

B16-SIY, MC38-AS, or EG7 cells in suspension were injected s.c. into mice on day 0. For the T cell adoptive transfer experiments, MC38-AS–bearing mice were sublethally irradiated with 600 cGy on day 7. MC38 tumor-reactive effector T cells were prepared as described previously (53). In brief, spleen-derived CD8^+^ T cells were isolated from MC38-bearing mice effectively immunized by irradiated MC38 tumor cell lysate–pulsed bone marrow–derived dendritic cells (DCs). These splenic T cells were cultured with tumor lysate–pulsed bone marrow–derived DCs for 5 days in vitro at a responder-to-stimulator ratio of 20:1. After 5 days of restimulation, cultures were resuspended and harvested, and placed in complete medium containing IL-2 at 20 U/mL and IL-7 at 5 ng/mL. Cell cultures were counted routinely and replenished with fresh complete medium and cytokines every 2–3 days. These cultures were restimulated twice with irradiated MC38 tumor cell lysate–pulsed DCs (at a 20:1 responder-to-stimulator ratio) every 5 days. After 15 days, the CD8^+^ T cells were harvested and tested for cytolytic activity, and cultured further in the presence of anti-CD3 or peptide, anti-CD28, 5′-AMP, and GSH with or without A2ARi, Lip-1, or both A2ARi and Lip-1 for 24 hours. On days 8 and 15 after tumor challenge, MC38 tumor-reactive CD8^+^ T cells were transferred i.v. at 2 × 10^6^ per mouse. Tumor-bearing mice were treated continuously daily i.p. with A2ARi (SCH58261) at 1 mg/kg for 2 weeks after transfer of ex vivo–treated T cells. Tumor volumes were measured along 3 orthogonal axes (*a*, *b*, and *c*) and calculated as *abc*/2 every 2–4 days. Tumor volume beyond 1,500 cm^3^ was regarded as end point, which was recorded for survival curve. For immunophenotyping of adoptively transferred CD8^+^ T cells, MC38-reactive CD8^+^ T cells were generated from CD90.1^+^ donor mice for ACT. OT-1 CD90.1^+^CD8^+^ T cells were treated ex vivo as above and infused i.v. into EG7-bearing mice (0.5 × 10^6^ to 1 × 10^6^ per mouse). One week after T cell transfer, the percentages and phenotype of transferred T cells in the tumor were determined by flow cytometry. For *Rag^–/–^* hosts, the percentages and phenotype of transferred T cells in the TME were determined by flow cytometry on day 16. For the rechallenge experiment, 1 × 10^6^ EG7 cells were inoculated into tumor-free hosts on day 90 or naive mice.

### Liquid chromatography–mass spectrometry metabolite analysis.

For in vitro T cell tracing experiments, isolated CD8^+^ T cells were activated and treated (as described in *In vitro cell treatment*, in Supplemental Methods) for 7 days. Treated T cells were counted in triplicate, resuspended in glucose-free growth RPMI medium with [^13^C-U]glucose (11 mM), and incubated for 4 hours at 37°C at 2 × 10^6^ viable cells per milliliter. For both metabolomics and tracing experiments, treated CD8^+^ T cells were collected and washed with cold saline solution twice in 15 mL tubes. After centrifugation at 300*g* for 5 minutes at 4°C, 1 mL of 80% cold methanol was added. The cell lysate/methanol mixture was transferred to a 1.5 mL conical tube and frozen in liquid nitrogen. The samples were subsequently subjected to 3 freeze-thaw cycles between liquid nitrogen and 37°C, with vertexing for 30 seconds after each thaw. The samples were again centrifuged at 20,000*g* for 15 minutes at 4°C. The supernatant was collected in new tubes and normalized by cell number. Samples were kept at −80°C until measured. The detection of intracellular metabolites in CD8^+^ T cells was performed at the Northwestern University Metabolomics Core Facility using liquid chromatography–mass spectrometry.

### Seahorse assay.

Activated CD8^+^ T cells were treated with vehicle, A2ARi, Lip-1, or a combination of both as described above, followed by further incubation in a hypoxia incubator chamber filled with hypoxic mixture containing 1.5% O_2_, 10% CO_2_, and 88.5% N_2_. One extra vehicle group in an atmosphere incubator served as the normoxia control. After 48 hours, treated T cells were harvested for the OCR measurement in an Extracellular Flux Analyzer XF96 (Seahorse Bioscience, North Billerica) with the Cell Mito Stress Test Kit. The measurements were conducted according to the manufacturer’s instructions.

### ChIP assay.

ChIP assay was performed with a ChIPAb+ E2F-1 kit (Millipore) following the manufacturer’s protocol. The cross-linking was performed with 1% paraformaldehyde for 10 minutes. Activated CD8^+^ T cells were lysed in SDS lysis buffer containing 1× protease inhibitor. Sonication was then performed, and protein/DNA complex was precipitated by antibody against E2F1 (Millipore) or IgG control (Millipore). DNA was purified using spin columns from the kit. ChIP-enriched chromatin was used for real-time PCR. Relative expression levels were normalized to input. The primers used for ChIP assay were: Gapdh: forward 5′-GCCCTTCTACAATGCCAAAG-3′, reverse 5′-TTGTGATGGGTGTGAACCAC-3′; GPX4: forward 5′-GCAACCAGTTTGGGAGGCAGGAG-3′, reverse 5′-CCTCCATGGGACCATAGCGCTTC-3′.

### Statistics.

Mean values were compared using an unpaired 2-tailed Student’s *t* test. When more than 2 groups were assessed, data were determined by 2-way ANOVA followed by Tukey’s or Šidák’s multiple-comparison tests. The statistical differences between the survivals of groups of mice were calculated according to the log-rank test. The correlations between 2 elements were calculated by Pearson’s correlation. Probability values greater than 0.05 were considered non-significant.

### Study approval.

All animal experiments were approved by Northwestern University’s Institutional Animal Care and Use Committee (IACUC).

### Data availability.

Bulk RNA sequence and ATAC sequence data were deposited to the NCBI’s Gene Expression Omnibus database with the data set identifier GSE190603. We used the SCP1039, SCP1288, SCP1493, SCP398, and SCP1162 data sets from the Broad Institute Single Cell Portal. Other data generated are available upon reasonable request. Values for data points shown in graphs and values behind means are reported in the Supporting Data Values file.

Further information is available in Supplemental Methods.

## Author contributions

SC and BZ conceived and designed the study. SC, JF, PX, and JA developed methodology. SC, JF, PX, MF, and LKB acquired data (provided animals, acquired and managed patients, provided facilities, etc.). SC, PX, JDW, JM, DAW, YW, YZ, NSC, and BZ analyzed and interpreted data (e.g., statistical analysis, biostatistics, computational analysis). SC, PX, NSC, JDW, DAW, YW, YZ, DF, JS, NSC, and BZ wrote, reviewed, and/or revised the manuscript. SC, JF, MF, and BZ provided administrative, technical, or material support (i.e., reporting or organizing data, constructing databases). BZ supervised the study.

## Supplementary Material

Supplemental data

Supporting data values

## Version 1 03/05/2024

In-Press Preview

## Version 2 04/15/2024

Electronic publication
