# Supplementary material for: CD8+ T cells sustain antitumor response by mediating crosstalk between adenosine A2A receptor and glutathione/GPX4
Source: J Clin Invest. 2024 Mar 5;134(8):e170071. doi: 10.1172/JCI170071 (PMC11014673; doi:10.1172/JCI170071)

**Supplemental Methods**

*Mice, cell lines, and reagents*. C57BL/6J, Rag1$^{-/-}$, Pmel, OT-I, *gpx4*$^{f/f}$, A2AR$^{f/f}$, VAV-cre, CD4-cre, CD8-cre, CD90.1 and NSG mice were purchased from the Jackson Laboratory. CD73$^{-/-}$ mice were generated by Dr. Linda Thompson (Oklahoma Medical Research Foundation, Oklahoma City). Our study examined male and female animals, and similar findings are reported for both sexes. All animal experiments were approved by institutional animal use committees of Northwestern University. Dr. Hans Schreiber (University of Chicago, Chicago) provided the 2C mice, EG7, CT26, B16F10 and B16-SIY cell lines. B16F10 cells were infected with MIGR1-ovalbumin (OVA)-IRES-eGFP19, and OVA-expressing cells (B16-OVA) were sorted based on GFP expression. OVA production was confirmed by ELISA. MC38-AS cell line was obtained from Dr. Arlene Sharpe (Harvard Medical School, Boston). LLC1 and HCC1806 cell lines were purchased from ATCC. All these cell lines were routinely tested for mycoplasma infections by culture and DNA stain and maintained in complete medium composed of RPMI 1640 with 5% FBS. CAR-T cells were purchased from ProMab and cultured in ImmunoCult™-XF T Cell Expansion Medium supplement with 25 μg/ml ImmunoCult™ Human CD3/CD28 T Cell Activator and 10 ng/ml human IL-2.

GSH/GSSG-Glo Assay Kit was purchased from Promega. SCH58261, adenosine 5'-monophosphate (5'-AMP) and cell permeable glutathione (glutathione ethyl ester) were purchased from Cayman Chemical Company. GSSG was purchased from Goldbio. Liproxstatin-1 and RSL-3 (1S,3R-) were purchased from MedChem Express. L-Buthionine-sulfoximine (BSO) was purchased from Sigma-Aldrich. Monobromobimane (mBBr) was purchased from Invitrogen. BODIPY 581/591 C11 (BODIPY), MitoTraker™ Green FM and MitoTracker™ Red CMXRos were purchased from Thermo Fisher Scientific. CGS 21680, H89 and HLM 006474 were from Tocris Bioscience. [U-$^{13}$C] glucose was purchased from Cambridge Isotope Laboratories.

Depleting mAb clone TY/23 (anti-CD73, BE0209) was purchased from BioXCell.

Antibodies for functional studies are ImmunoCult™ Human CD3/CD28 T Cell Activator (STEMCELL Technologies, Catalog No. 10971, Working concentration 25ug/ml), anti-mouse CD3 (Clone 145-2C11, BioLegend, Catalog No. 100360, Working concentration: 1μg/mL) and anti-mouse CD28 (Clone 37.51, BioLegend, Catalog No. 102121, Working concentration: 1μg/mL).

Antibodies for FACS are anti-mouse CD8 (53-6.7, BioLegend, Catalog No. 100712, 100708 and 100706), anti-mouse IFN-γ (XMG1.2, BioLegend, Catalog No. 505826 and 505808), anti-mouse TNF-α (MP6-XT22, BioLegend, Catalog No. 506333), anti-mouse GranzymeB (GB11, BioLegend, Catalog No. 515408), anti-mouse Ki67 (11F6, BioLegend, Catalog No. 151212), anti-Annexin V (BioLegend, Catalog No.640941 ), 7-AAD (BioLegend, Catalog No. 420404), anti-mouse CD90.1 (OX-7, BioLegend, Catalog No. 202516), anti-mouse Vα2 (B20.1, BioLegend, Catalog No. 127810), anti-mouse CD45 (30-F11, BioLegend, Catalog No. 103108), anti-mouse CD3 (145-2C11, BioLegend, Catalog No. 100328), anti-mouse Lag-3 (C9B7W, BioLegend, Catalog No. 125227), anti-mouse CD69 (H1.2F3, BioLegend, Catalog No. 104512), anti-mouse PD-1 (J43, BD, Catalog No. 565815), anti-mouse Ly108 (13G3, BD, Catalog No. 742272), anti-mouse GCLC (EPR20078, Abcam, Catalog No. ab207777) and anti-mouse GPX4 (EPNCIR144, Abcam, Catalog No. ab125066).

Antibodies for immunoblot and ChIP are anti-mouse GSR (Thermo Scientific, Catalog No. PA5-79334, Working concentration: 1:10), anti-mouse PKA C-α (Cell Signaling Technology, Catalog No. 4782S, Working concentration: 1:50) and anti-mouse E2F1 (KH20 and KH95, Millipore, Catalog No. 17-10061).

Antibodies for mIHC are anti-human Ki67 (Abcam, Catalog No. ab15580, Working concentration 1:500, Assigned fluorophore Opal 520), anti-human GCLC (ThermoFisher, Catalog No. MA5-32850, Working concentration 1:2000, Assigned fluorophore Opal 540), anti-human CD8 (Cell signaling, Catalog No. 70306S, Working concentration 1:200, Assigned fluorophore Opal 570), anti-human GPX4 (ThermoFisher, Catalog No. PA5-102521, Working concentration 1:500, Assigned fluorophore Opal 620), anti-human A2AR (Sigma, Catalog No. HPA075997, Working concentration 1:200, Assigned fluorophore Opal 650) and anti-human PanCK (Abcam, Catalog No. ab27988, Working concentration 1:500, Assigned fluorophore Opal 690).

*Analysis of cells by flow cytometry*. All samples were initially incubated with 2.4G2 to block antibody binding to Fc receptors. Single-cell suspensions were stained with 1 μg of relevant mAbs and then washed twice with cold PBS. For cell apoptosis analysis, cells were rinsed with 1x binding buffer, and then stained with Annexin V and 7AAD along with other surface antibodies in binding buffer at RT in the dark for 10 mins and immediately run on a flow cytometer. Ki67 staining was performed according to the manufacturer's instructions (eBioscience). Intracellular IFN-γ, TNF-α and granzyme B staining under stimulation with 50 ng/ml PMA, 5 μg/ml ionomycin plus 10 μg/ml BFA in the presense of the relevant tumor antigen peptides, was performed according to the manufacturer's instruction (BD Bioscience). For BODIPY and mBBr staining, wash cells with 2 ml of PBS and centrifuge at 300g for 5 min at RT. Resuspend the cells in 0.5 ml of PBS prewarmed to 37 °C. Add BODIPY at the concentration of 5uM and gently vortex. Incubate the cell suspension for 20 min at 37 °C. Then add mBBr at the concentration of 50uM and incubate the cell suspension for 10 min at 37 °C. Wash cells with 1 ml of PBS and centrifuge at 300g for 5 min at 4 °C. For measurement of mitochondrial membrane potential and mass, $CD8^+$ T cells were stained with 200 nM MitoTracker Red CMXRos (MTR) and 100 nM MitoTracker Green FM (MTG) for 15 min at 37 °C. Samples were harvested on LSRII and FACSCanto, and data were analyzed with FlowJo software. The gating strategy was shown in Supplemental Figure 12.

*RNA extraction and real-time PCR*. Total RNA was extracted using Trizol reagent (Invitrogen) according to the manufacturer's instructions. The cDNA synthesis was performed using SuperScript One-Step RT-PCR (Invitrogen). Quantitative real-time PCR was used to quantify genes by SYBR Green (Bio-Rad), and relative abundance of each mRNA was normalized to GAPDH mRNA. The primers used for q-RT-PCR are: GAPDH:F: 5'-ACCACAGTCCATGCCATCAC-3', R: 5'-TCCACCACCCTGTTGCTGTA-3'; GPX4: F: 5′-GCAACCAGTTTGGGAGGCAGGAG-3′, R: 5′-CCTCCATGGGACCATAGCGCTTC-3′; GCLC: F: 5'-AGAACACGGGAGGAGAGAGG-3', R: 5'-CTTACTGATCCTAAAGCGATTGTTC-3'; GCLM: F: 5'-GACTCACAATGACCCGAAAGA-3', R: 5'-GATGCTTTCTTGAAGAGCTTCCT-3'; GSTO1: F: 5' - CTAAGGTGCCGCCTTTGA - 3', R: 5' - CTCCTTGAGCTCCAATGCTT - 3'; E2F1: F: 5'-GGATCTGGAGACTGACCATCAG-3', R: 5'-GGTTTCATAGCGTGACTTCTCCC-3'.

*In vitro cell treatment*. $CD8^+$ T cells were purified from WT, $A2AR^{f/f}$ VAV-cre, $GPX4^{f/f}$ CD4-cre, OT1-$GPX4^{f/f}$ CD4-cre, OT1, 2C or Pmel mice. On day 0, anti-CD3 (1 μg/ml) or peptide OVA-I (10 ng/ml) or SIY (0.5 μg/ml) or gp100 (0.5 μg/ml), anti-CD28 (1 μg/ml), 5'-AMP (50 μM), GSH (500 μM) were added with/without A2ARi (1 μM), Lip-1 (5 μM) or A2ARi/Lip-1. On day 1, cells were washed and suspended with fresh medium in the presence of anti-CD3 or peptide, anti-CD28 and 5'-AMP. Additional experiments were conducted using a lesser TCR activation input such as

anti-CD3 (0.5 μg/ml)/anti-CD28 (0.5 μg/ml), or peptide OVA-I (1 μg/ml) for OT-1 cells with/without 5'-AMP, GSH, A2ARi, or Lip-1 as above. For Neon electroporation transfection (Thermofisher), cells were resuspended in T buffer from the kit and mixed with Piggybac transposon reagents and 10 μg Gpx4- or Gclc-overexpressed plasmid. Apply electrical pulse to the cells with Neon program #24 (1600 V/ 10ms/ 3 pulses). On day 4, cells were split and added with fresh medium, anti-CD3 or peptide, anti-CD28 and 5'-AMP. On day 7, cells were counted and analyzed for T cell apoptosis, cytokine production and proliferation by flow cytometry.

*EM for mitochondria morphology*. Activated $CD8^+$ T cells were washed with PBS and gently resuspended in fixative buffer (3% glutaraldehyde, 2% formaldehyde in 0.1 M PIPES, pH 7.2) for 1 hour at RT. Samples were analyzed in Northwestern University NUANCE's BioCryo facility. The number of mitochondria per cell was counted. For mitochondrial cristae, the crista number or total length in one mitochondrion was calculated from high-magnitude EM images of live cells.

*Immunoblotting*. Activated $CD8^+$ T were washed and lysed in RIPA lysis buffer with 1× protease inhibitor cocktail (Millipore Sigma). The lysates were obtained and stored at −80 °C until immunoblot analysis. The quantification of lysates was performed by BCA assay following manufacturer's protocol (Thermo Fisher Scientific). The samples were loaded in the Jess Simple Western platform (Bio-Techne/proteinsimple) for protein detection and quantification following manufacturer's protocol. The protein bands were visualized and quantified in the Compass Software (Bio-Techne/proteinsimple).

*Bulk RNA sequence*. RNA from activated $CD8^+$ T cells was extracted using Direct-zol RNA Miniprep kit (Zymo Research) as described by the manufacturer's protocol. The RNA of each sample was quantified using the Qubit RNA HS Assay Kit (Thermo Fisher Scientific), and RNA integrity was evaluated by 2100 Bioanalyzer (Agilent Technologies). mRNA was isolated using the NEBNext Poly(A) mRNA Magnetic Isolation Module (New England Biolabs), and cDNA libraries were generated using the low-input strand-specific RNA-Seq kits NEBNext Ultra II RNA Library Prep Kit for Illumina (96 reactions) and NEBNext Multiplex Oligos for Illumina (96 index primers) (New England Biolabs). All RNA and DNA purification steps were performed using AMPure XP Beads (Beckman Coulter Inc.). The quantity and quality of cDNA were assessed using the Qubit dsDNA HS Assay Kit (Thermo Fisher Scientific). Libraries were sequenced at Northwestern University Sequencing Core (NUSeq) in the Illumina HiSeq 4000 platform using single-end 50-bp reads and generating average 20 million reads per sample.

*ATAC sequence*. The tagmentation and purification of DNA from activated $CD8^+$ T cells was completed following the manufacturer's protocol (Acitve Motif). Tagmented DNA was amplified using PCR with a combination of one i7 Indexed Primer and one i5 Indexed Primer provided by manufacturer. The DNA clean-up step was performed by using AMPure XP Beads (Beckman Coulter Inc.). The quantity and quality of DNA libraries were assessed using the DNA/RNA Qubit Measurement and Bioanalyzer (DNA 1000 assay). Libraries were sequenced at Northwestern University Sequencing Core (NUSeq) in the Illumina NextSeq 500 platform using paired-end 37.5-bp reads and generating average 25 million reads per sample.

*Multiplex immunohistochemistry (mIHC)*. The breast carcinoma tissue microarray (TMA) containing invasive ductal carcinoma cases (IDC, Grade 2, n=28, Grade 3, n=22), medullary

carcinoma cases (MCB, n=9) and adjacent normal breast tissue (normal, n=3) was purchased from US Biomax Inc, and utilized for mIHC staining of CD8, A2AR, Ki67, GPX4, GCLC, PanCK and DAPI followed the instruction of the Opal 7-Color IHC kit (AKOYA Biosciences) as described previously (1, 2). Briefly, the TMA was baked at 65°C for one hour, deparaffinized with xylene, re-hydrolyzed in sequential decreased concentrations of ethanol and re-fixed in 10% neutral buffered formalin prior to antigen retrieval in AR9 retrieval buffer (AKOYA Biosciences) using a high-pressure antigen retrieval oven for 15 minutes. Afterwards, the TMA was implemented six rounds of staining procedures, including blocking, incubation of primary antibody, HRP-labeled second antibody and developed with the assigned Opal fluorophore. The TMA was heated in AR6 retrieval buffer using the high-pressure oven for 15 minutes at the end of each round staining to release the bounded primary and second antibodies, while undisturbed the developed Opal fluorophore signal. After six-round staining procedures, the slide was counterstained with DAPI (AKOYA biosciences), and mounted with Diamond anti-fade fluorescence mounting media (Thermo Fisher Scientific). The single marker staining with individual opal fluorophore was served as a reference of the spectral library for the "spectral unmixing process", and the unstained slide was served as the background control.

*Acquirement of multispectral images (MSI) and data analysis*. The Opal fluorophore signals from individual biopsies on TMA or the single Opal reference staining slide were captured using the Vectra 3 Automated Quantitative Pathology Imaging System (Perkin Elmer) at high magnification (20x) through five emission spectral filters including DAPI, FITC, Cy3, Texas Red and Cy5. By using InForm Advanced Image Analysis software (Akoya Biosciences), the captured images were proceeded with spectral unmixing into seven individual fluorophores based on the unique emitting spectrum of each single staining fluorophore. Subsequently, the unmixed images underwent tissue segmentation into tumor-nest and stroma according to tumor epithelial marker PanCK, cell segmentation based on membrane or cytoplasm specific markers and DAPI and cell phenotyping based on specific cellular markers. The data containing composite images, tissue segmentation, cell segmentation and cell phenotyping from InForm were exported for further quantitative analyses of cellular densities and protein intensities using R-based phenoptrReports & phenoptr (AKOYA biosciences).

Supplemental Figure 1

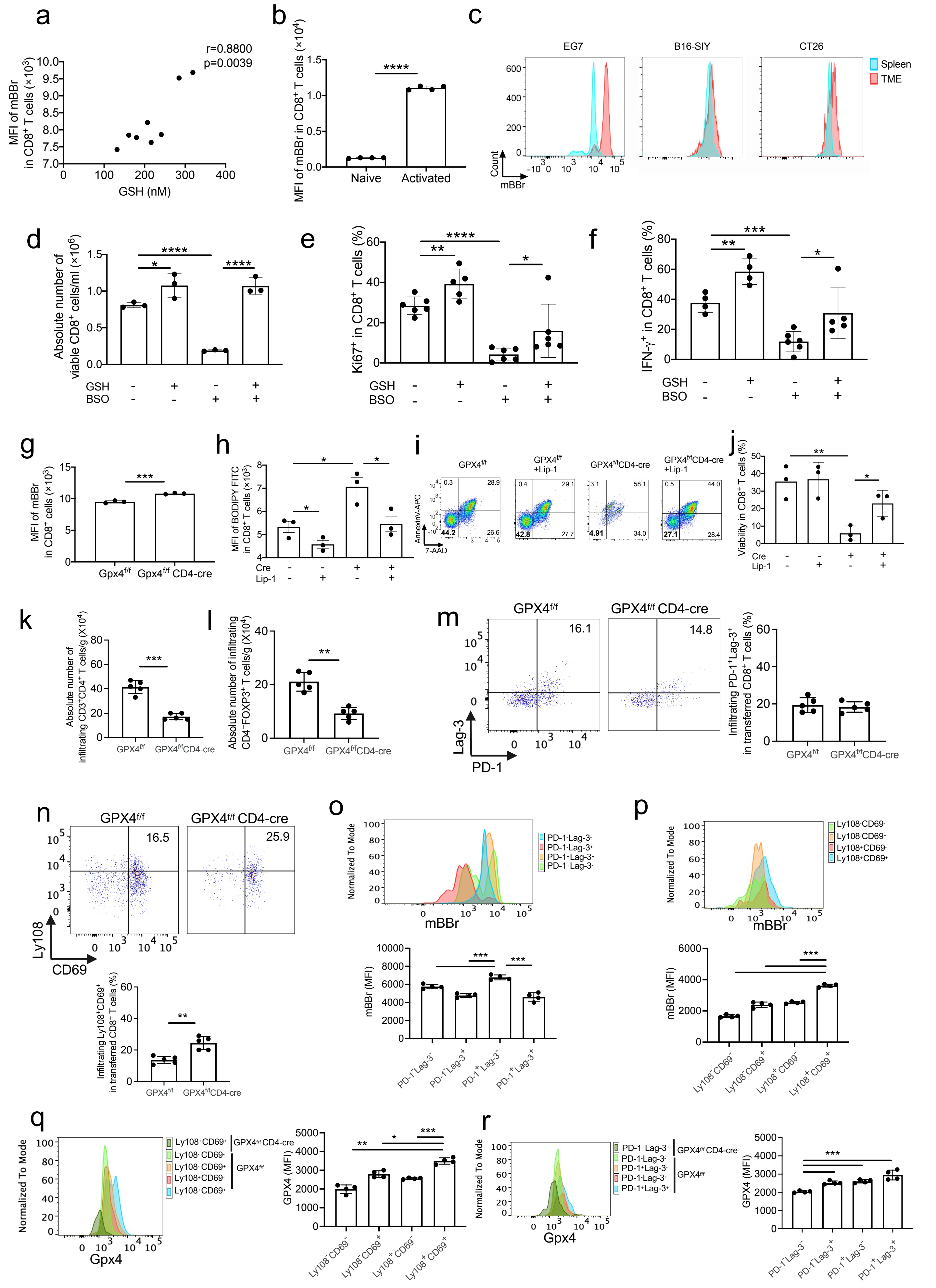

**Supplemental Figure 1. GSH availability is required for activated CD8$^+$ T cell responses.**

**a**, The correlation between MFI of mBBr and intracellular GSH measured by GSH/GSSG-Glo Assay in activated CD8$^+$ T cells. **b**, Intracellular GSH was measured by mBBr in OT1 CD8$^+$ T cells treated with or without 10 ng/ml OVA-I and 1 μg/ml anti-CD28. **c**, Intracellular GSH content indicated by mBBr staining was measured in splenic CD8$^+$ cells and tumor infiltrating CD8$^+$ and CD45$^-$ cells from EG7, B16-SIY and CT26 bearing mice. **d-f,** OT1 CD8$^+$ T cells in the presence of 10 ng/ml OVA-I and 1μg/ml anti-CD28 were treated with or without 50 μM BSO, 500 μM cell permeable GSH or a combination of both. On day 7, the percentages and/or numbers of viable (**d**), Ki67$^+$ (**e**) and IFN-γ$^+$ (**f**) cells among activated OT1 CD8$^+$ T cells were measured. **g-j**, CD8$^+$ T cells from *Gpx4$^{f/f}$* and *Gpx4$^{f/f}$ CD4$^{cre}$* cKO mice in the presence of 10 ng/ml OVA-I and 1 μg/ml anti-CD28 were treated with or without 5 μM liproxstatin-1 (Lip-1). GSH content by mBBr (**g**), lipid ROS by BODIPY-C11 (**h**) and viability (**i, j**) were measured by flow cytometry. **k, l**, *Gpx4$^{f/f}$* and *Gpx4$^{f/f}$ CD4$^{cre}$* hosts were inoculated with MC38 tumor cells (n=5). On day 21, the MC38 tumors were harvested and tumor-infiltrating CD4$^+$ T cells (**k**) and CD4$^+$Foxp3$^+$ Tregs were calculated (**l**). **m-r**, Activated *Gpx4$^{f/f}$* or *Gpx4$^{f/f}$ CD4$^{cre}$* OT1 CD90.1$^+$CD8$^+$ T cells were *i.v.* transferred into EG7-bearing mice. One week after T cell transfer, the percentage of PD-1$^+$Lag-3$^+$(**m**)**, or** Ly108$^+$CD69$^+$ cells (**n**) in transferred CD90.1$^+$CD8$^+$ T cells among tumor infiltrates by flow cytometry. In parallel, the intracellular GSH levels as indicated by mBBr MFI were determined in different subsets related to exhausted-like state (**o**) or stem-like state (**p**) among infiltrating transferred CD90.1$^+$CD8$^+$ T cells by flow cytometry. The GPX4 expression levels (MFI) were also determined in different subsets related to exhausted-like state (**p**) or stem-like state (**q**) among infiltrating transferred CD90.1$^+$CD8$^+$ T cells by flow cytometry. Data were analyzed by the Pearson correlation (**a**) or two-tailed *t*-test (**b, k, g, l, n**), and two-way ANOVA (**d-f, h, j, o-r**) . Data plotted are mean ± s.e.m. $^*P < 0.05$, $^{**}P < 0.01$, $^{***}P < 0.001$, $^{****}P < 0.0001$.

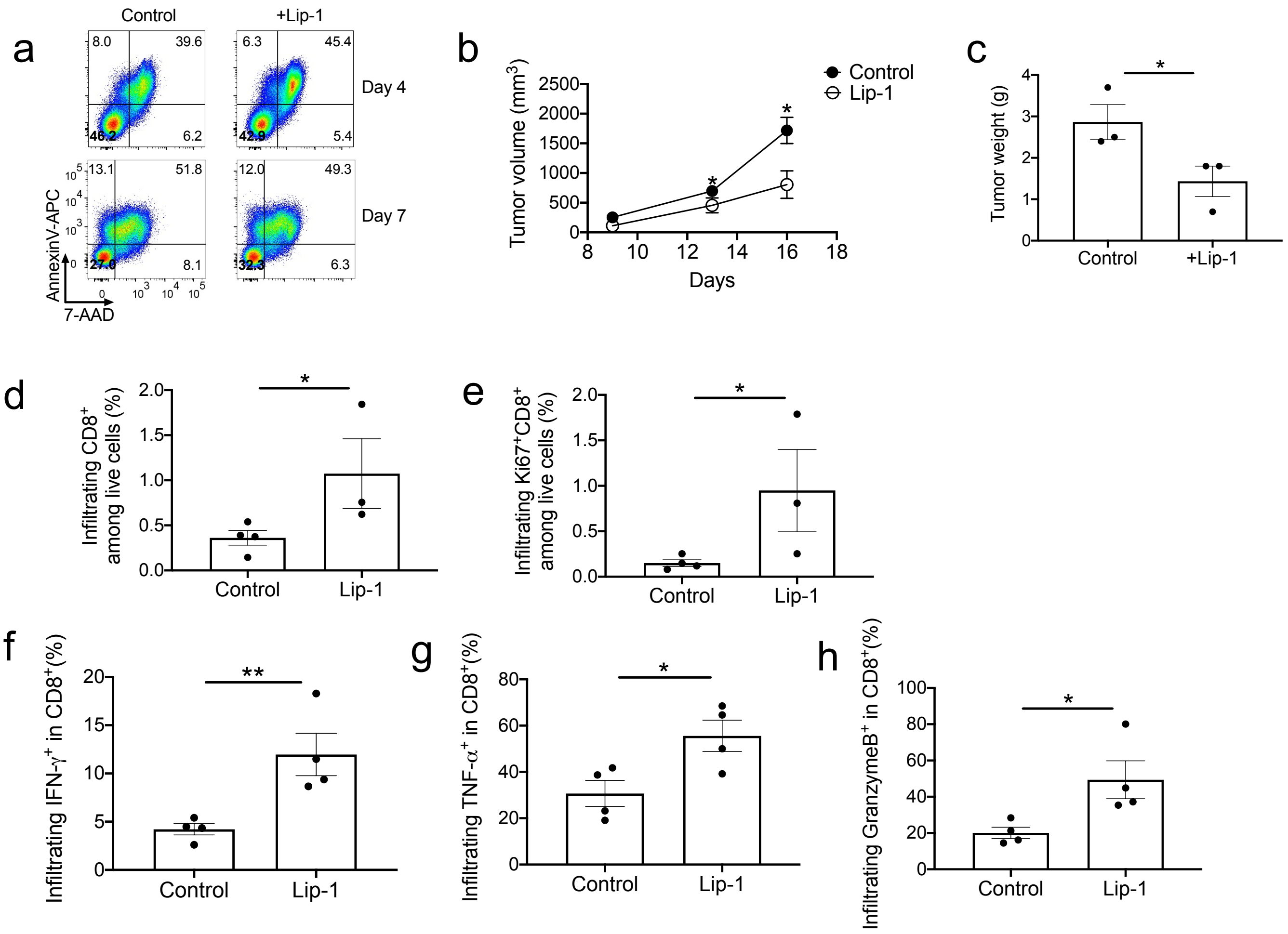


**Supplemental Figure 2. Activated CD8+ T cells treated with Liproxstatin-1 (Lip-1) *ex vivo* display superior anti-tumor activity**

**a,** Activated 2C CD8+ T cells in the presence of 0.5 μg/ml SIY and 1μg/ml anti-CD28 were treated with or without Lip-1. On day 4 and day 7, the viability of T cells was detected by flow cytometry. **b-h,** B16-SIY bearing $Rag^{-/-}$ hosts were transferred with 2 million activated 2C CD90.1+ CD8+ T cells treated with or without Lip-1. Tumor volume (**b**) and weight (**c**) were measured. Tumor-infiltrating transferred CD8+CD90.1+ cells were evaluated by flow cytometry (**d-h**). Data were analyzed by two-tailed *t*-test. Data plotted are mean ± s.e.m from biological replicates. $^{*}P < 0.05$, $^{**}P < 0.01$.

Supplemental Figure 3

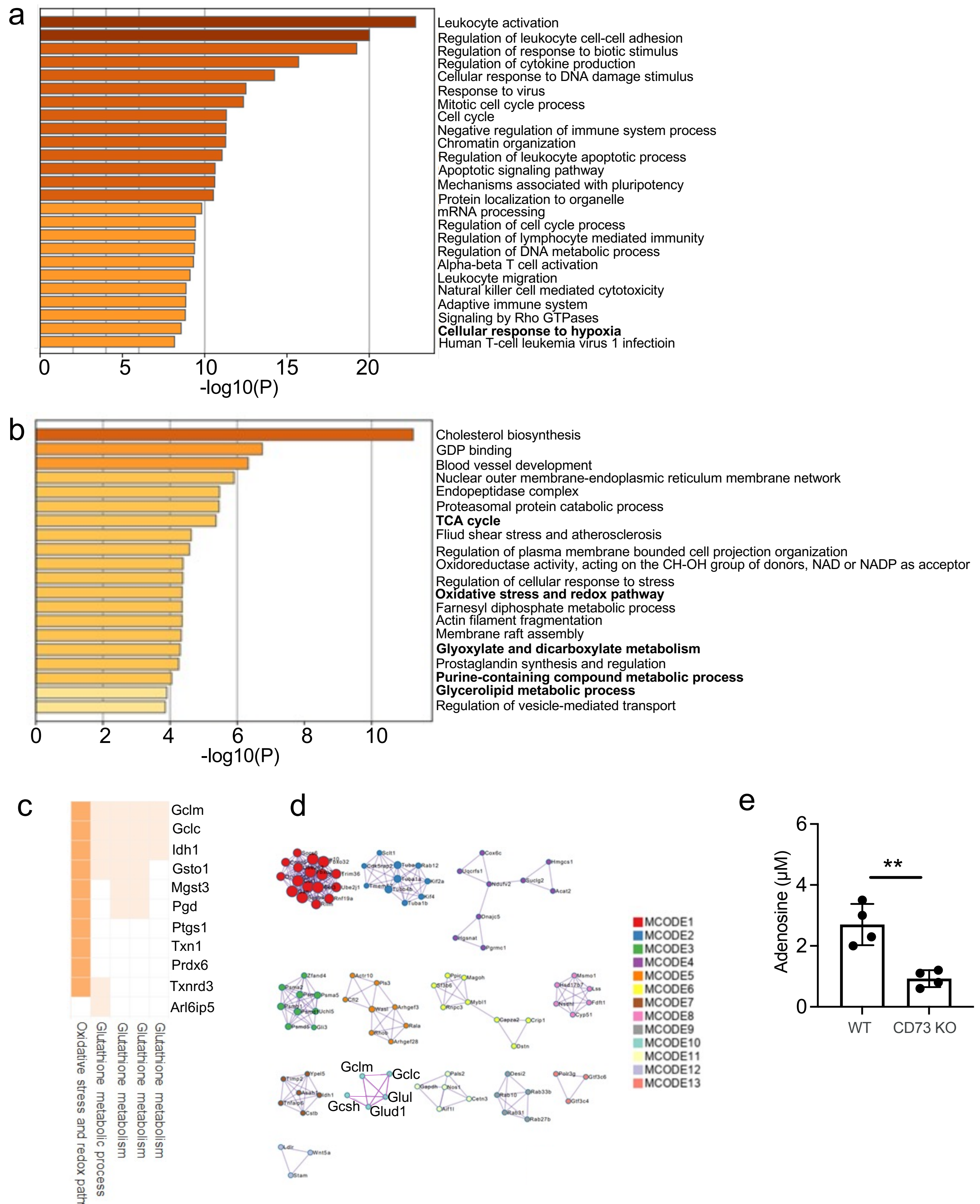


**Supplemental Figure 3. Adenosine pathway is related to essential cellular metabolism pathways.**

**a,** RNA-seq was performed in activated CD8+ T cells treated with or without Lip-1 *in vitro*. The enriched pathway was shown. **b-d,** RNA-seq was performed in enriched infiltrating CD8+ T cells from E0771-bearing mice treated with or without anti-CD73 (CD73i). Enriched pathways (**b**), heatmap of oxidative stress and redox pathway related genes (**c**) and gene clusters (**d**) were shown. **e,** Extracellular adenosine level was measured by HPLC from the culturing medium of WT or CD73 KO CD8+ T cells activated with anti-CD3 and anti-CD28 in the presence of 5'-AMP for 7 d *in vitro* (n=4). Data plotted are mean ± s.e.m. Data were analyzed by two-tailed *t*-test (**e**). $^{**}P < 0.01$.

Supplemental Figure 4

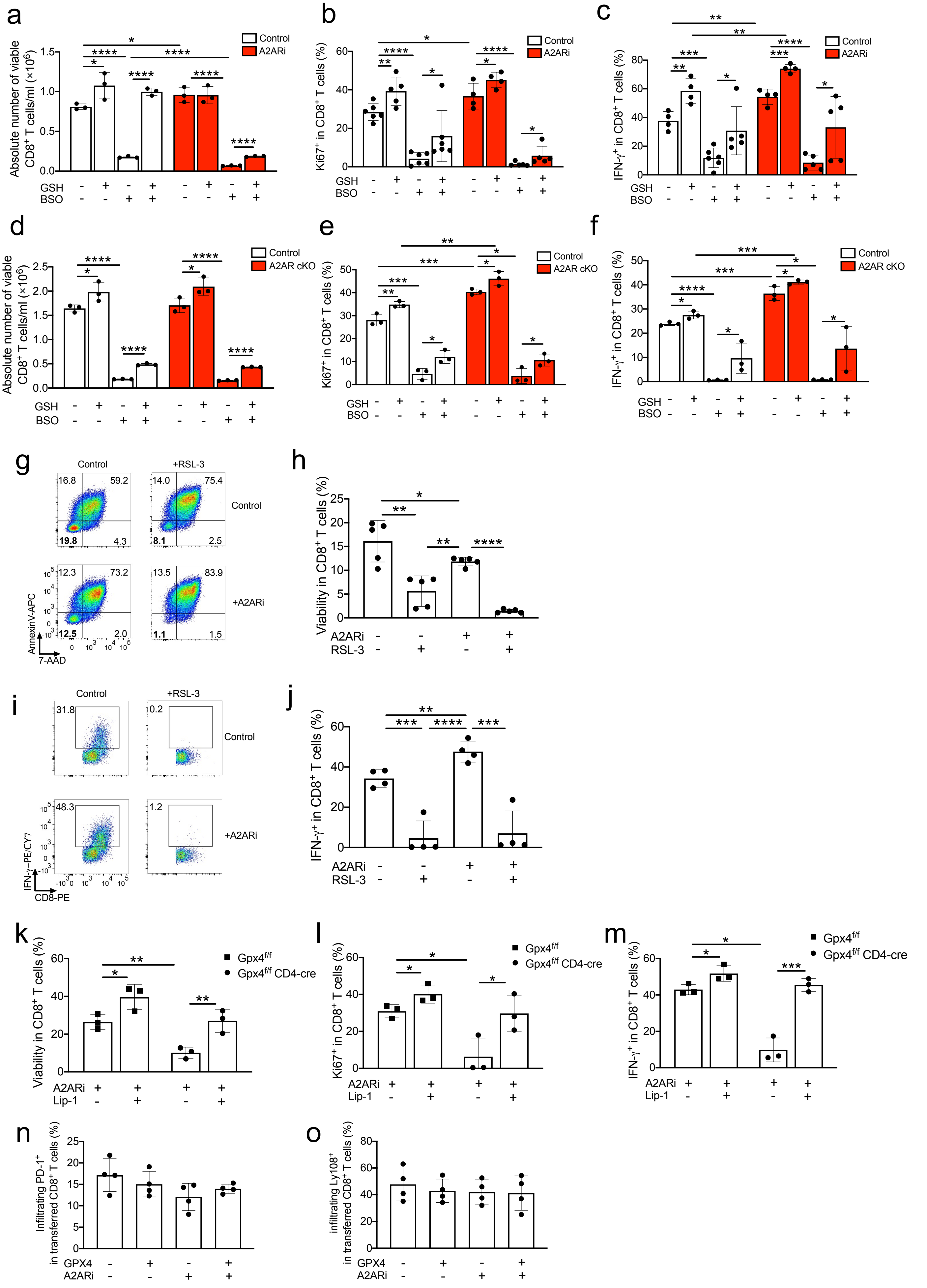

**Supplemental Figure 4. Inactivation of A2AR signaling orchestrates CD8$^+$ T cell responses by coordinating the GSH-Gpx4 axis**.

**a-c,** OT1 CD8$^+$ T cells from WT were activated by OVA-I and anti-CD28 in the presence of 5'-AMP with or without GSH, A2ARi, BSO or combination. After 7 days, the percentages and/or numbers of viable (**a**), Ki67$^+$ (**b**) and IFN-$\gamma^+$ (**c**) T cells were measured. **d-f,** CD8$^+$ T cells from WT or *A2AR$^{f/f}$ Lck$^{cre}$* cKO were activated by anti-CD3 and anti-CD28 in the presence of 5'-AMP with or without GSH, BSO or combination. After 7 days, the percentages and/or numbers of viable (**d**), Ki67$^+$ (**e**) and IFN-$\gamma^+$ (**f**) T cells were measured. **g-j**, Activated OT1 CD8$^+$ T cells in the presence of OVA-I, anti-CD28 and GSH were treated with or without A2ARi, Gpx4 inhibitor RSL-3 or a combination of both. After 7 days, the viable cells (**g, h**) and IFN-$\gamma^+$ T cells (**i, j**) were measured. **k-m,** *Gpx4$^{f/f}$* and *Gpx4$^{f/f}$ CD4$^{cre}$* OT1 CD8$^+$ T cells were activated by OVA-I and anti-CD28 in the presence of 5'-AMP and GSH with or without A2ARi or A2ARi+Lip-1. After 7 days, the percentages and/or numbers of viable (**k**), Ki67$^+$ (**l**) and IFN-$\gamma^+$ (**m**) T cells were measured. **n, o** MC38-bearing mice received sublethal irradiation on day 8 and were transferred i.v. with activated CD90.1$^+$CD8$^+$ T cells with or without ectopic GPX4 expression, treated with or without A2ARi or combination (n=4). 14 days after T cell transfer, the percentages of exhaustion marker PD-1$^+$ cells (**n**) and stem-like marker Ly108$^+$ cells (**o**) were measured in transferred CD8$^+$ T cells within tumors by flow cytometry. Results are representative of three independent experiments. Results are representative of more than three independent experiments. Data were analyzed by two-way ANOVA (**a-f, h, j, k-m**). Data plotted are mean ± s.e.m from biological replicates. $^*P < 0.05$, $^{**}P < 0.01$, $^{***}P < 0.001$, $^{****}P < 0.0001$.

Supplemental Figure 5

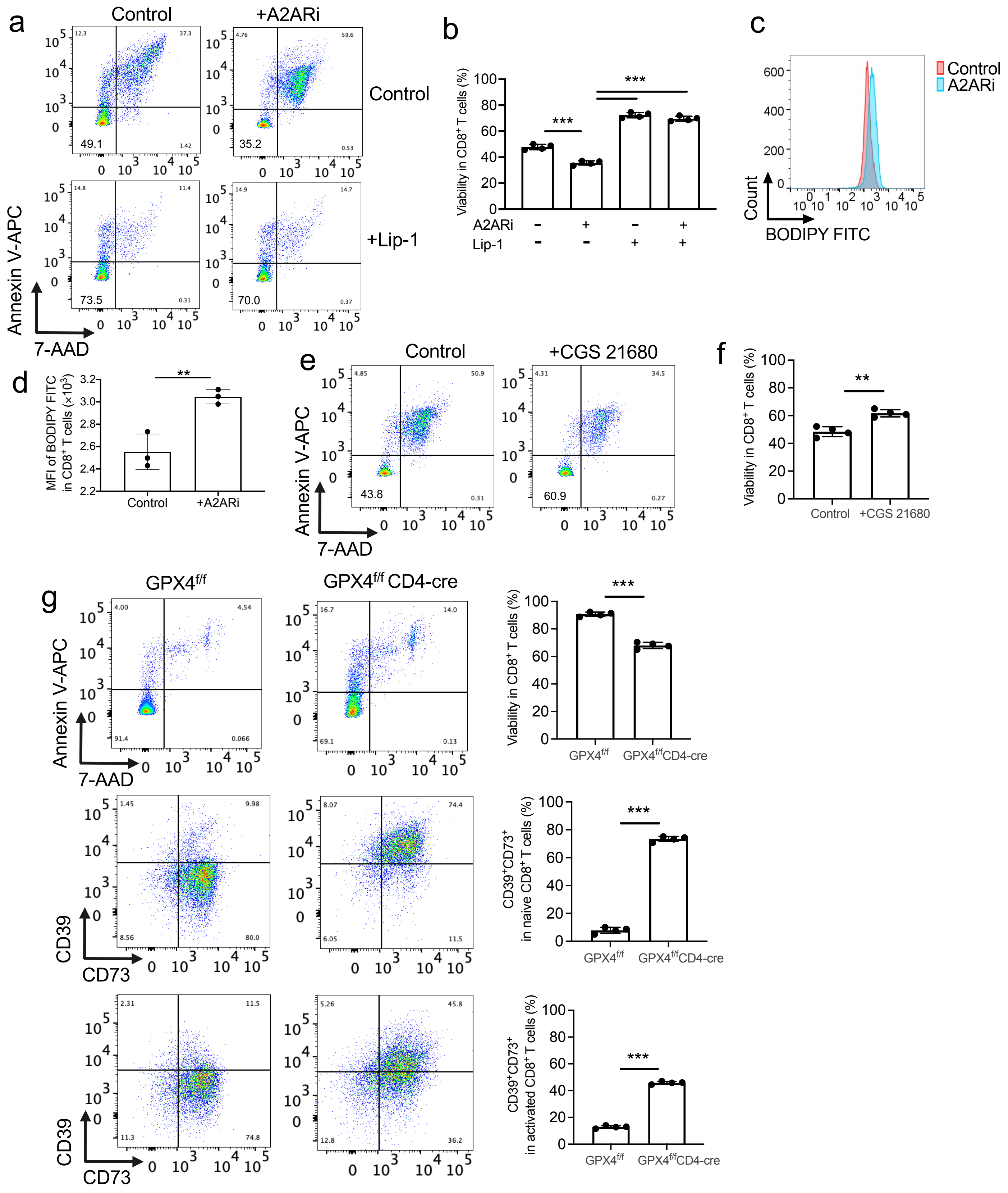


**Supplemental Figure 5. Inactivation of A2AR triggers ferroptosis in activated CD8⁺ T cells.**

**a-f,** Activated WT CD8⁺ T cells in the presence of anti-CD3, anti-CD28, IL-2 and 500 μM GSH were treated with or without A2ARi, Lip-1, A2ARi/Lip-1 (**a-d**) or A2AR agonist CGS 21680 (**e, f**). After 7 days, the viability and lipid ROS indicated by oxidized BODIPY-C11 staining were analyzed in these activated CD8⁺ T cells by flow cytometry (**c, d**). **g,** Activated splenic CD8⁺ T cells from *Gpx4f/f* and *Gpx4f/f CD4cre* cKO mice in the absence (naive) or presence of anti-CD3, anti-CD28, IL-2 and 500 μM GSH for 7 days. Viability of naive CD8⁺ T cells (upper), the percentage of CD73+CD39+ cells in naïve (middle) and activated (bottom) CD8⁺ T cells was determined by flow cytometry. Results are representative of more than three independent experiments. Data were analyzed by two-way ANOVA (**b**), and two-tailed *t*-test (**d, f, g**). Data plotted are mean ± s.e.m. $^{**}P < 0.01$, $^{****}P < 0.0001$.

Supplemental Figure 6

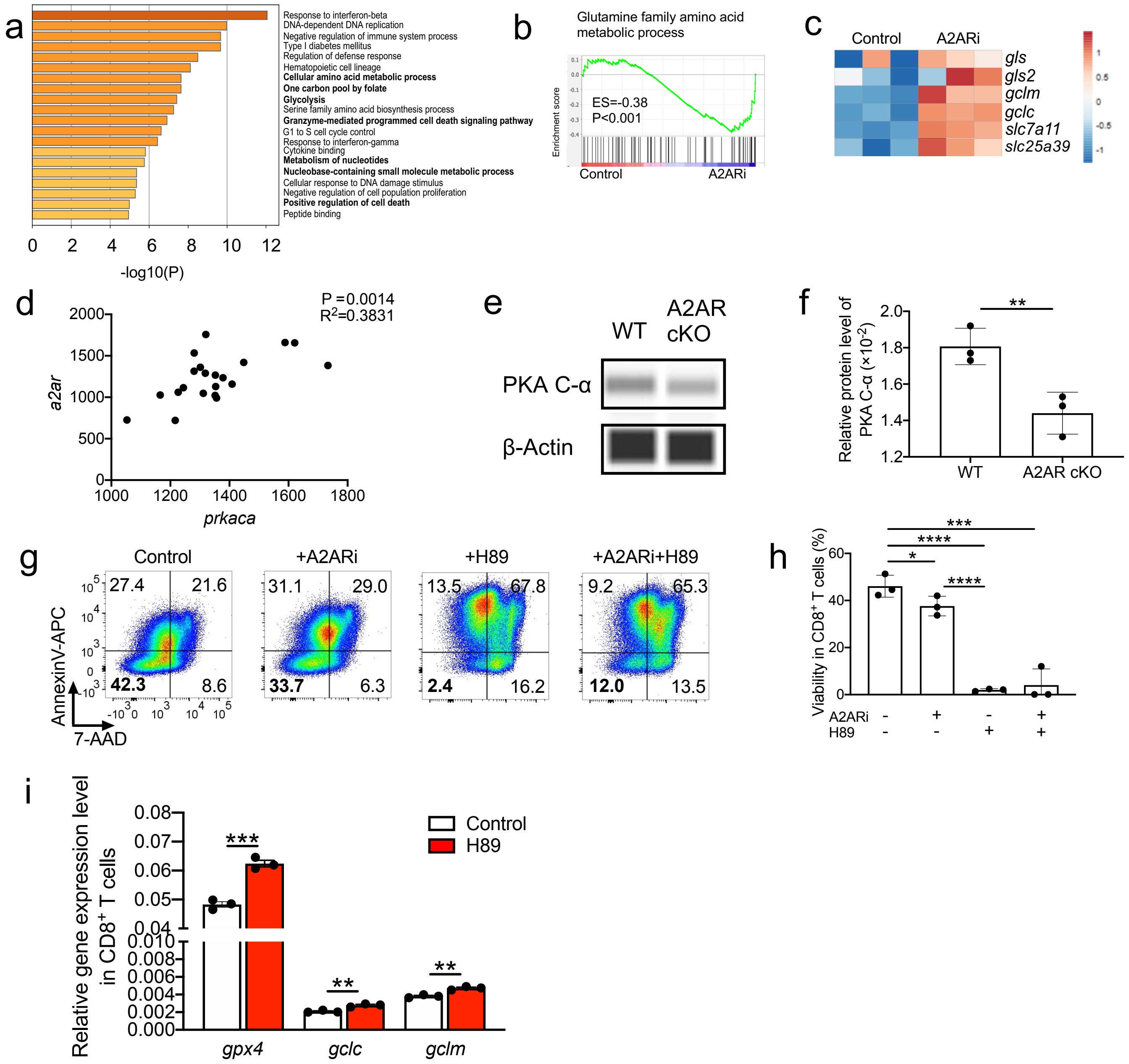


**Supplemental Figure 6. A2AR downstream signaling transcriptionally regulates the GSH metabolic pathway.**

**a-c**, RNA-seq was performed in activated Pmel CD8+ T cells treated with or without A2ARi. The pathway enrichment (**a**) and GSEA plot (**b**) showing the enriched pathway of glutamine amino acid metabolic process and the heatmap (**c**) showing enriched genes that particularly drive the *de novo* GSH biosynthesis and the mitochondrial GSH-import machinery. **d,** The correlation between gene expression level of *a2ar* and *prkaca* by RNA-seq in activated CD8+ T cells. **e, f,** Western blot analysis of PKA C-α expression in WT and *A2AR^f/f^ Lck^cre^* cKO CD8+ T cells. **g-i,** Activated OT1 CD8+ T cells in the presence of OVA-I, anti-CD28 and GSH were treated with or without 5 μM PKA inhibitor H89. After 7 days, the viable cells were analyzed by flow cytometry (**g, h**). After 1 day, the expression levels of *gpx4, gclc* and *gclm* in activated CD8+ T cells were evaluated by qRT-PCR (**i**). Data were analyzed by the Pearson correlation (**d**) or two-tailed *t*-test (**f, h, i**). Data plotted are mean ± s.e.m from biological replicates. $^*P < 0.05$, $^{**}P < 0.01$, $^{***}P < 0.001$, $^{****}P < 0.0001$.

Supplemental Figure 7

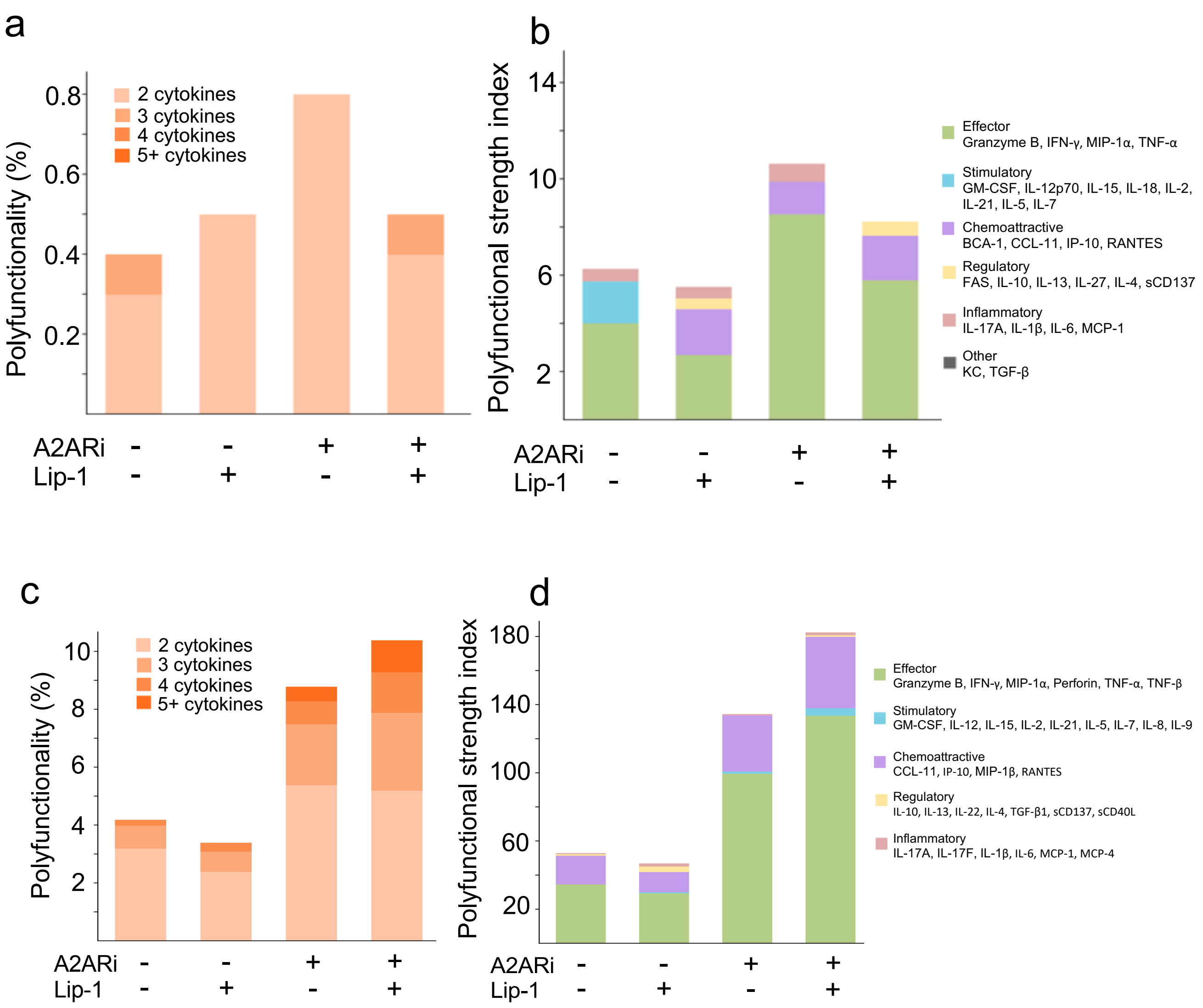


**Supplemental Figure 7. Combination of Lip-1 and A2ARi augments polyfunction of activated CD8⁺ T cells.**

**a, b**, Activated WT CD8⁺ T cells in the presence of anti-CD3, anti-CD28 and IL-2 were treated with or without Lip-1, A2ARi or a combination of both. The polyfunctionality (**a**) and polyfunctional strength index (**b**) indicated by relative abundance and composition of secreted cytokines at single cell level were measured by IsoPlexis IsoLight assays using Isoplexis Mouse Adaptive Immune Chips. **c, d**, Anti-MSLN CAR-T cells were activated *in vitro* by anti-CD3, anti-CD28 and IL-2 with or without Lip-1, A2ARi or combination. The polyfunctionality (**c**) and polyfunctional strength index (**d**) indicated by relative abundance and composition of secreted cytokines at single cell level were measured by IsoPlexis IsoLight assays using Isoplexis Human Adaptive Immune Chips.

Supplemental Figure 8

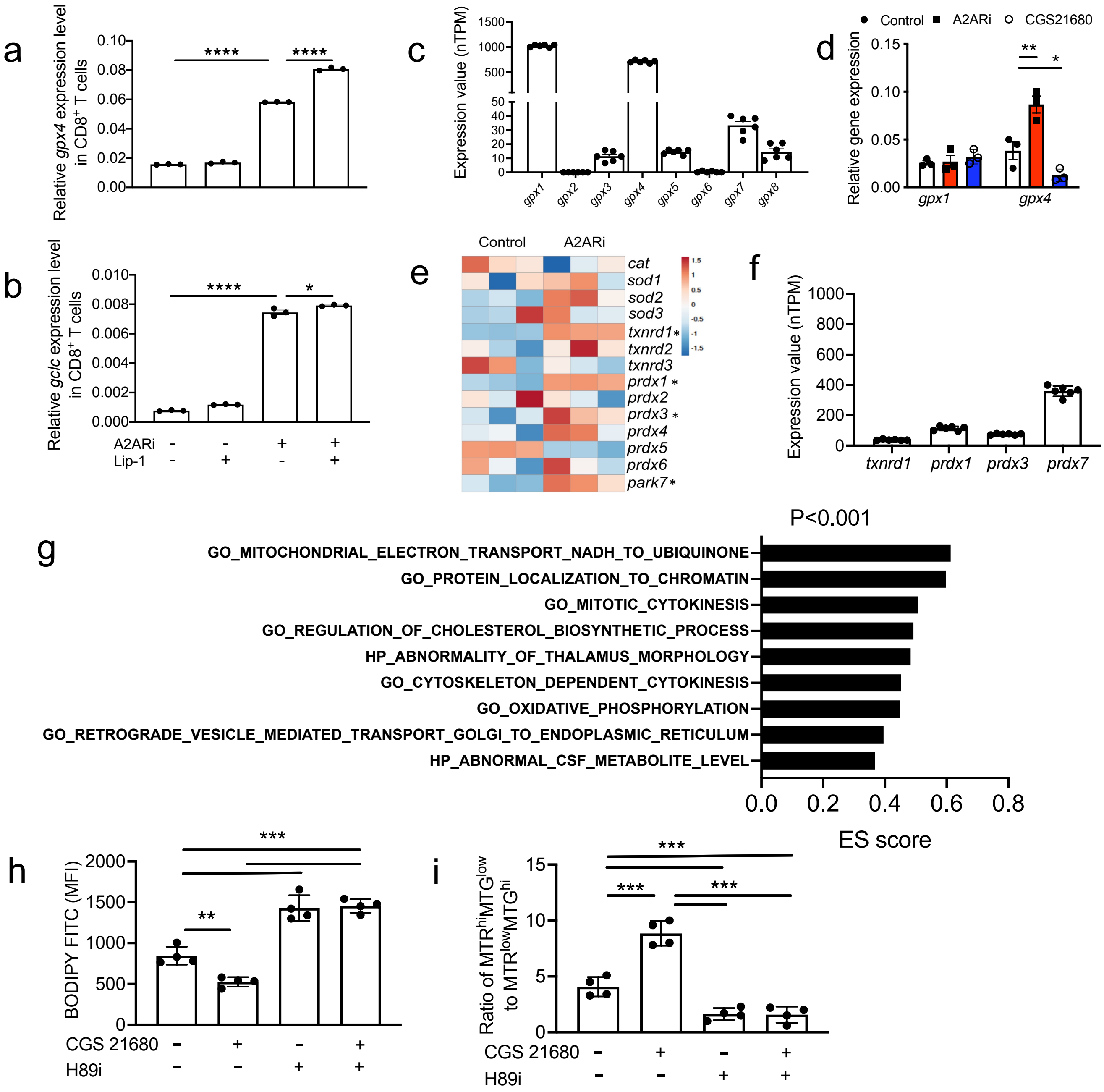

**Supplemental Figure 8. Combination of both A2ARi and Lip-1 reveals distinct metabolic changes from the transcriptomic profiling of activated CD8⁺ T cells.**

**a, b,** The qRT-PCR was performed to detect expression levels of *gpx4* (**a**) and *gclc* (**b**) in activated Pmel $CD8^+$ T cells treated with or without A2ARi, Lip-1 or a combination of both. **c,** Expression levels (nTPM) of *gpx* family members obtained from Bulk-RNA-seq analysis of in vitro-activated $CD8^+$ T cells. **d,** qRT-PCR analysis of expression levels of *gpx1* and *gpx4* in activated Pmel $CD8^+$ T cells treated with or without A2ARi, or CGS 21680. **e, f,** Expression levels (nTPM) of key genes related to antioxidant enzymes obtained from Bulk-RNA-seq analysis of in vitro-activated $CD8^+$ T cells. **g,** Activated OT1 $CD8^+$ T cells in the presence of OVA-I, anti-CD28 and GSH were treated with or without Lip-1, A2ARi or a combination of both. RNA-seq was performed in activated T cells and GSEA analysis showed the overlapped enriched pathway in a combination of both compared to single treatment alone. **h, i,** Activated OT1 $CD8^+$ T cells in the presence of OVA-I, anti-CD28 and GSH were treated with or without CGS 21680, H89i or a combination of both. After 7 days, the BODIPY FITC signal for detection of lipid ROS (**h**), and the ratio of $MTR^{hi}MTG^{low}$ to $MTR^{low}MTG^{hi}$ (**i**) was calculated based on flow analysis. Results are representative of three independent experiments. Data were analyzed by two-tailed *t*-test. Data plotted are mean ± s.e.m. Data were analyzed by two-way ANOVA (**a, b, h, i**), an $*P < 0.05$, $**P < 0.01$, $***P < 0.001$, $****P < 0.0001$.

Supplemental Figure 9

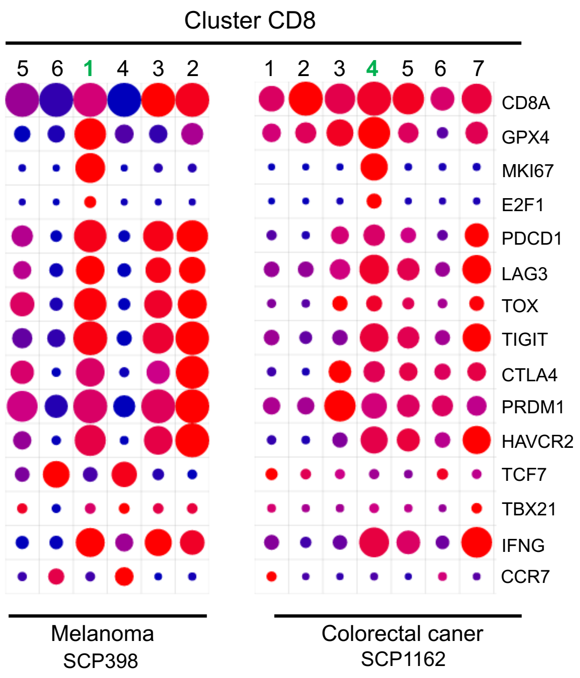


**Supplemental Figure 9. Distinct gene signatures in the tumor-infiltrating GMSG CD8+ T cell cluster from conventional exhausted-like, or stem-like T cells .**

Dot plot of scRNA-seq data (https://singlecell.broadinstitute.org/single_cell) showing geometric mean expression (log(TP10K+1)) and frequency (dot size) of key genes associated with exhausted, stem-like and memory phenotypes in clusters of tumor-infiltrating CD8$^{+}$ T cells from patients with melanoma (n=59) and colon cancer (n=62)**.**

Supplemental Figure 10

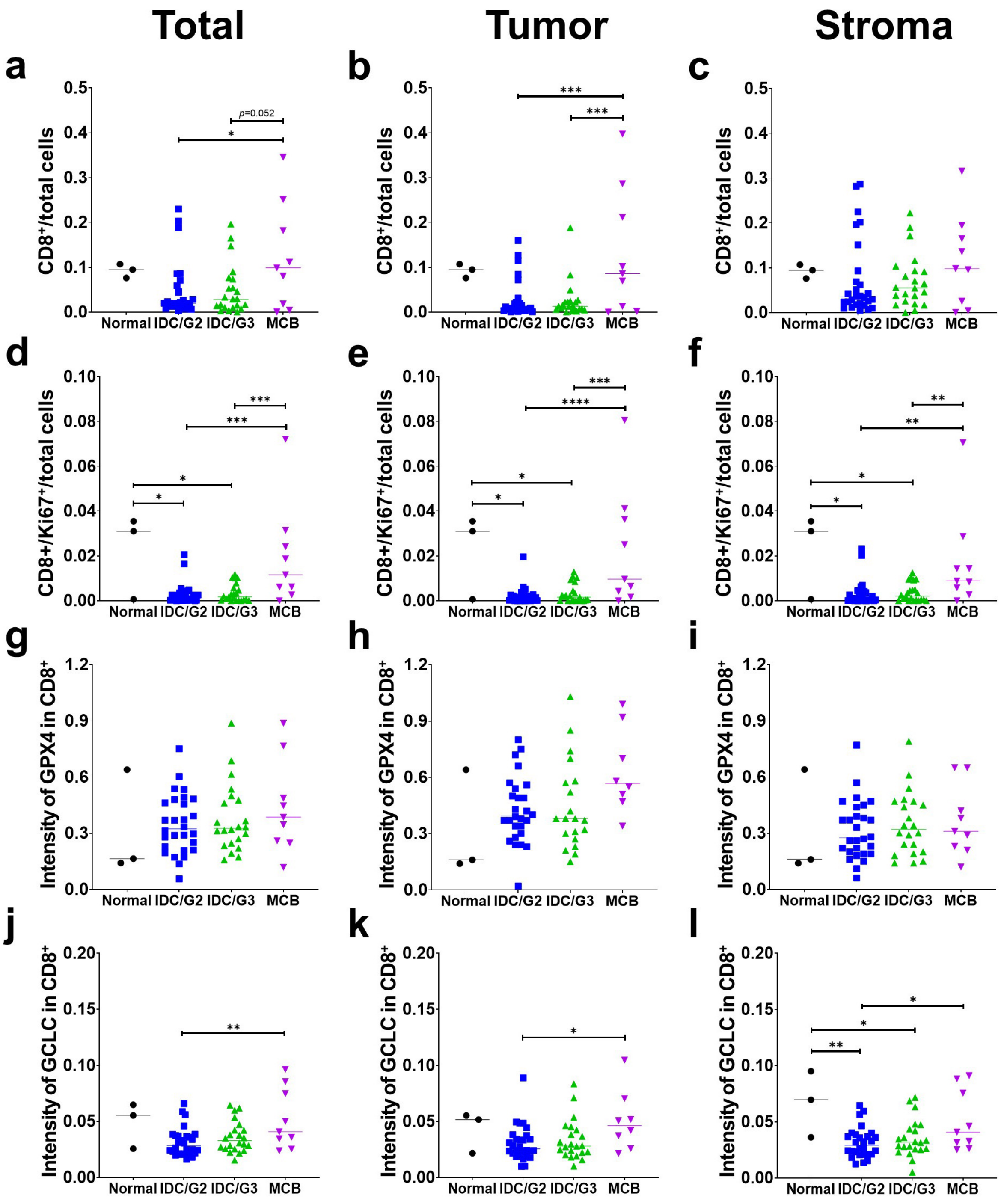


**Supplemental Figure 10. Characterization of tumor-infiltrating CD8+ T cells in human breast cancers by mIHC.**

The density of $CD8^+$ (**a-c**) or $CD8^+ki67^+$ (**d-f**) was compared between cases with invasive ductal carcinoma (IDC, G2; n=27; G3; n=24), medullary carcinoma of the breast (MCB, n=9) and normal breast tissues (n=3) in total tissue, tumor-nest compartment or stroma compartment. The intensity of Gpx4 (**g-i**) or Gclc (**j-l**) was compared between cases with IDC (G2; n=27; G3; n=24), MCB (n=9) and normal breast tissues (n=3) in total tissue, tumor compartment or stroma compartment. Data were analyzed by two-way ANOVA. $^*P < 0.05$, $^{**}P < 0.01$, $^{***}P < 0.001$, $^{****}P < 0.0001$.

Supplemental Figure 11

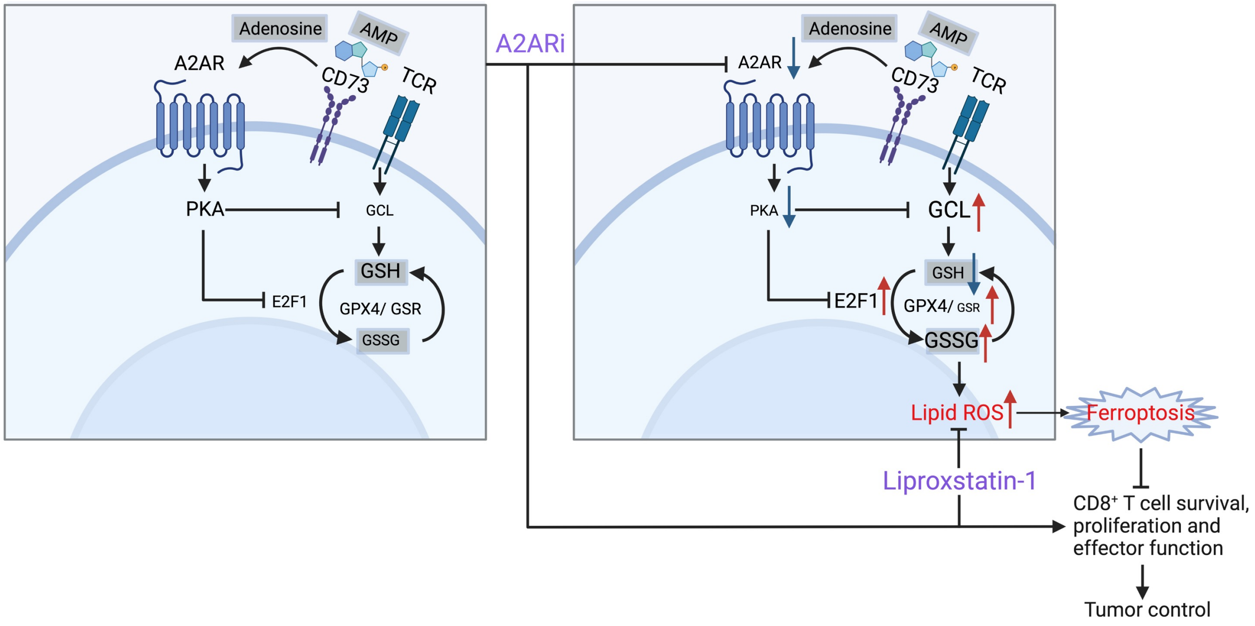


**Supplemental Figure 11. The proposed mechanism underlying an antitumor response of CD8+ T cells by modulating GSH metabolism and the A2AR signaling.**

# Supplemental Figure 12. Gating strategy

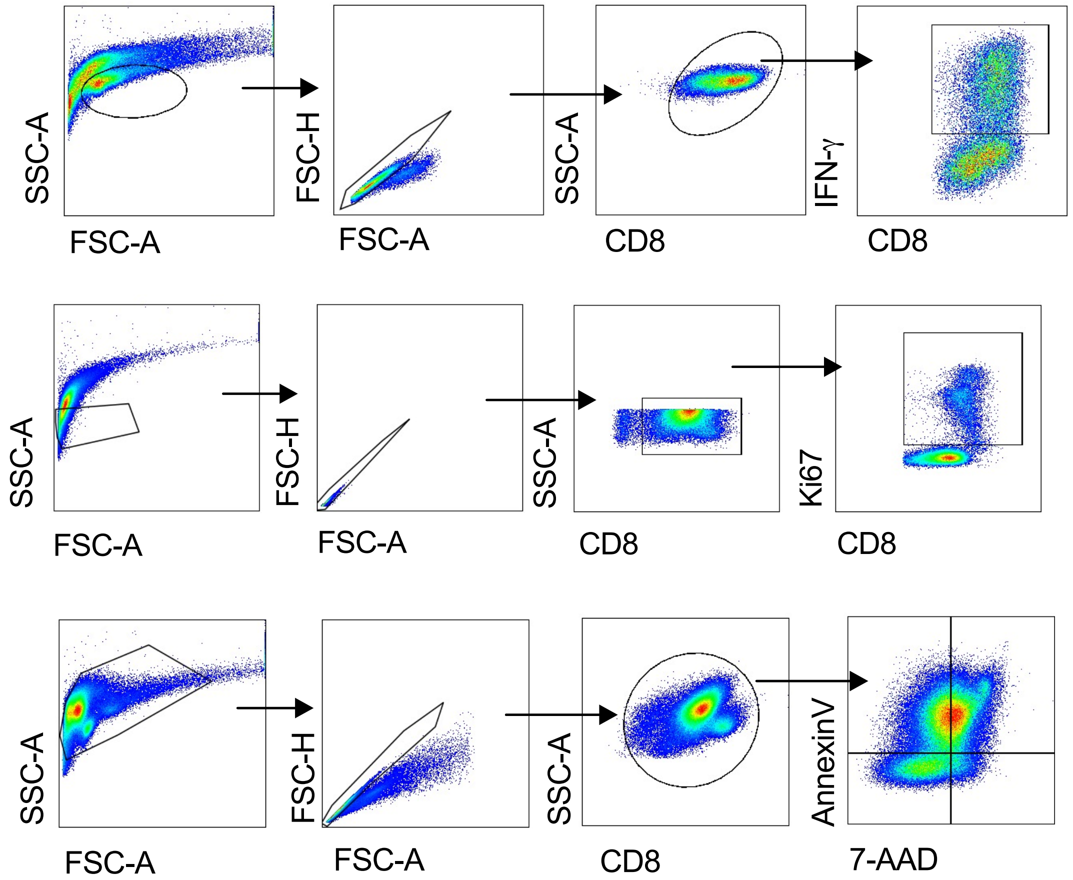


## Immunophenotyping of tumor-infiltrating T cells

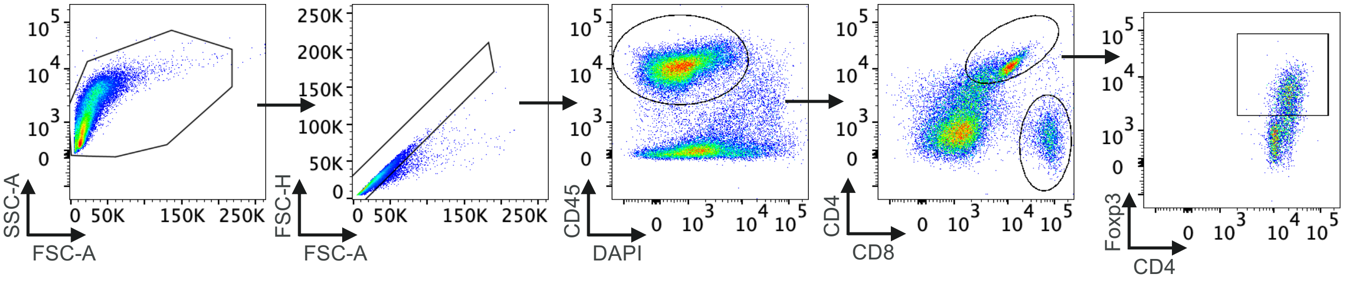


## Immunophenotyping of adoptively transferred tumor-infiltrating CD8⁺ T cells

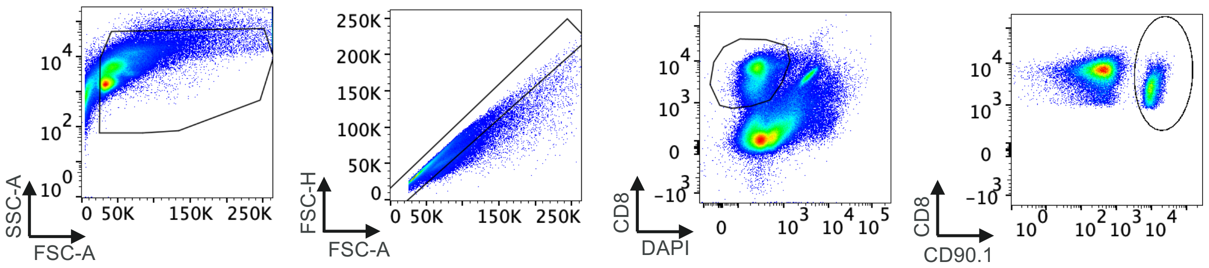

Supplement: Supplemental data [file jci-134-170071-s024.pdf]
